# So Pathogenic or So What?—A Brief Overview of SIV Pathogenesis with an Emphasis on Cure Research

**DOI:** 10.3390/v14010135

**Published:** 2022-01-12

**Authors:** Adam J. Kleinman, Ivona Pandrea, Cristian Apetrei

**Affiliations:** 1 Division of Infectious Diseases, DOM, School of Medicine, University of Pittsburgh, Pittsburgh, PA 15261, USA; adk68@pitt.edu; 2 Department of Infectious Diseases and Immunology, School of Public Health, University of Pittsburgh, Pittsburgh, PA 15261, USA; pandrea@pitt.edu; 3 Department of Pathology, School of Medicine, University of Pittsburgh, Pittsburgh, PA 15261, USA

**Keywords:** human immunodeficiency virus (HIV), simian immunodeficiency virus (SIV), latency reversing agents (LRAs), HIV latency, reactivation, pathogenesis, cure, strategies, nonhuman primate models

## Abstract

HIV infection requires lifelong antiretroviral therapy (ART) to control disease progression. Although ART has greatly extended the life expectancy of persons living with HIV (PWH), PWH nonetheless suffer from an increase in AIDS-related and non-AIDS related comorbidities resulting from HIV pathogenesis. Thus, an HIV cure is imperative to improve the quality of life of PWH. In this review, we discuss the origins of various SIV strains utilized in cure and comorbidity research as well as their respective animal species used. We briefly detail the life cycle of HIV and describe the pathogenesis of HIV/SIV and the integral role of chronic immune activation and inflammation on disease progression and comorbidities, with comparisons between pathogenic infections and nonpathogenic infections that occur in natural hosts of SIVs. We further discuss the various HIV cure strategies being explored with an emphasis on immunological therapies and “shock and kill”.

In 1985, shortly after the first discovery of HIV-1 [1], a group at the New England Regional Primate Research Center (NEPRC) reported the identification of a nonhuman primate (NHP) lentivirus counterpart of HIV-1 (that would later be known as SIVmac), which was responsible for AIDS cases in the rhesus macaques (*Macaca mulatta*, RMs) colony of the NEPRC [2]. SIVmac, specifically clone SIVmac239 and viral swarm SIVmac251, would become the gold standard for nonhuman primate cure modeling. As discussed later, SIVmac greatly recapitulates many aspects of HIV pathogenesis. Regardless, curing HIV has become a substantial challenge. Antiretroviral therapy (ART) is one of the greatest medical miracles of the last few decades, being able to drastically suppress HIV replication and incredibly extend the life expectancy of persons living with HIV (PWH) [3]. However, ART is virostatic and does not directly eliminate infected cells or proviruses from PWH and the aging PWH population is suffering from increased comorbidities, leading to a decreased quality of life and an increase in healthcare costs [4,5,6]. Additionally, through decades of cure research, only two PWH have been demonstrated to achieve complete HIV remission: the “Berlin patient” and the “London patient”. These two individuals were treated with stem cell transplantations from donors homozygous for the *CCR5 ∆32* allele for their cancers [7,8]. However, this strategy is neither scalable nor does it have acceptable toxicity for the vast majority of PWH. In this review, we discuss the origins of SIV strains used in research and the roles of various SIV-nonhuman primate models, as well as the pathogenesis of SIV/HIV and current strategies utilized in HIV cure.

## 1. You Can Call Me SIV: Introduction and the Origin of SIVmac

In the early 1970s, an outbreak of lymphomas, resembling Burkitt’s lymphoma, was reported in RMs housed at the California National Primate Research Center (CNPRC) [9,10,11,12] and would later be demonstrated to play a role in the SIVmac infections at NEPRC. However, the origin of these pathogenic lentiviruses in the RMs remained unknown then, as studies in the wild macaques from Asia did not identify any circulation of SIV-like viruses in these NHP species [13,14,15]. Meanwhile, a plethora of SIVs was shown to naturally infect multiple species of monkeys and apes in Africa [13]. These viruses are highly divergent from each other and show a diversity profile evocative of host-dependent evolution [16,17], suggesting a very old origin of SIVs, predating the monkey speciation in Africa [18]. Yet, the fact that no New World monkeys carry SIVs, nor are the Old World monkeys in Asia, points to an origin of the AIDS viruses sometime after the speciation of the Asian monkeys [18]. Interestingly, the virus isolated from the macaques in NEPRC and CNPRC was closely related to the SIV naturally infecting sooty mangabeys (*Cercocebus atys, SM*) [19].

**Figure 1:**
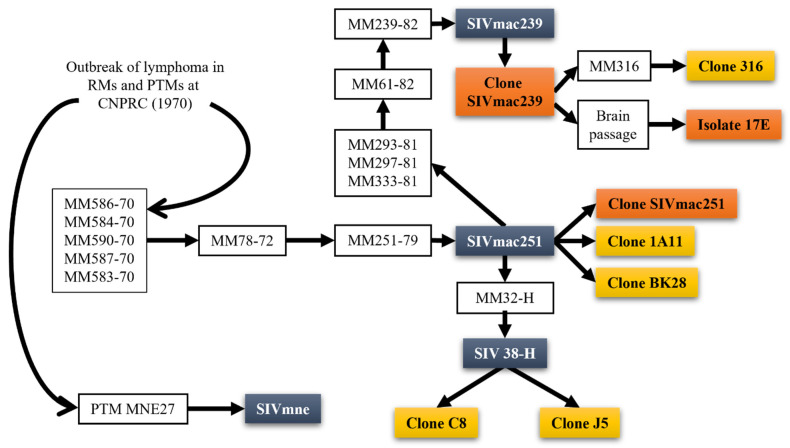
Origin of SIVmac251, SIVmac239, and derivative clones. SIVmac239 and SIVmac251 originate from rhesus macaques housed at the California National Primate Research Center (CNPRC). The progenitor viruses were from sooty mangabeys at the CNPRC which were used for kuru experiments, allowing for serial passaging and eventual establishment of the SIVmac239 and SIVmac251 isolates. White boxes are animals and passages; bluish grey boxes are the primary strains recovered from passaging; orange boxes are the clones most used in research; yellow boxes are lesser used clones.

As such, the origin of the SIV infection in captive macaques at the NEPRC was perplexing, particularly when applying the criteria of the cross-species transmission that allowed the identification of the sources of HIVs in chimps and gorillas from Cameroon for HIV-1 [20] and in SMs from West Africa for HIV-2, respectively [21]: (a) genetic, antigenic and phylogenetic similarities between the human and NHP viruses; (b) coincidence between the species habitat and the HIV-1/HIV-2 epicenters; (c) favoring factors of transmission. These requirements were largely not fulfilled for the origin of the SIVmac in the macaque colony at the NEPRC, as the NEPRC did not have any SM. Meanwhile, at the CNPRC, both RMs and SMs were housed at the same time in the 1960s, yet, reports suggested that the two species did not enter in close direct or indirect contact [11]. In a twist of events, however, virus archeology studies performed at the NEPRC clearly demonstrated that the origin of SIVmac was in fact at the CNPRC, from survivors of the original lymphoma outbreak that were shipped to the NEPRC in the 1970s (Figure 1) [22,23]. The virus then went undetected for >10 years in the NEPRC colony. The proofs of the virus transfer are: (i) detection of SIV antibodies in the CRPRC RMs with lymphomas; (ii) pathologies observed were similar to what is now known as pathogenic SIV infection; (iii) detection of SIV antibodies in the SMs in the CRPRC colony prior to the outbreak RM exposure to sooty mangabey tissues; (iv) detection of SIVmac DNA in the spleen and lymph nodes in one of the RMs sent to NERPRC [23]. More recently, extensive phylogenetic analyses of the SIVs naturally infecting SMs from different Primate Centers in the US traced the origin of the SIVmac to SMs in the CNPRC [22]. Moreover, the circumstances of the accidental transmission from SMs to RMs were established to rely on the kuru experiments carried out extensively at the CNPRC and New Iberia Research Center (NIRC) in the 1960s [19]. These experiments by D. Carleton Gadjdusek initially passaged human brain extracts into SMs to try to discover the cause of kuru after he discovered the disease in New Guinea. SM brain extracts were then serially passaged into RMs, allowing for direct transmission of SIV.

Furthermore, studies have shown [22] that the same experiments carried out at the CNPRC were likely responsible for the infection of SIVs of different other species of macaques, such as the pigtailed macaques (*Macaca nemestrina*) [24] and the stumptailed macaque (*Macaca arctoides*) [25,26] and crab eating macaque (*Macaca fascicularis*) [27].

## 2. Why Don’t You Infect Me? The Animal Model for AIDS Research

There are multiple advantages of the use of the NHP model for AIDS research. The most important of these is that animal studies allows us to perform interventions that would otherwise be impossible to perform in PWH: staged infections, invasive sampling, exploratory interruptions of antiretroviral therapies, testing of new therapeutic approaches and vaccines. The model has been extensively characterized over the last three decades, and a wealth of data is available for comparisons. Moreover, multiple virological, immunological and clinical biomarkers have been extensively tested and developed, conferring the model predictability and consistency. It is therefore not surprising that the NHP models for AIDS research, which recapitulate the key features of HIV infection, provided seminal results for HIV prevention, pathogenesis, and treatment.

The early events of HIV transmission and dissemination in the host, with the potential impact on prevention and treatment were obtained in NHPs and showed a very rapid seeding of the reservoirs [28,29]. Further, the use of NHP models has provided seminal information regarding the persistence of this reservoir and acts as an excellent tool for screening new strategies aimed at inducing cure/functional cure [28,30,31,32,33,34,35,36]. For example, studies showed that the major site of virus replication and CD4^+^ T-cell depletion is at the mucosal sites, pointing to the mucosa as the major target of vaccine interventions for the prevention of HIV transmission [37,38,39,40] and these studies predated those in PWH study participants by a decade. Further, because of the ease of manipulation in NHPs, it has been shown that diet can have a large effect on disease progression such that high fat diets accelerate progression [41]. Similarly, experimental infections allow for a better understanding of transmission and the differences between routes of infection [42,43,44].

The comparison between natural hosts of various SIV strains, which do not progress to AIDS, have played a big role in understanding pathogenesis. Studies on the cartography of viral dissemination [45] pointed to major differences between pathogenic and nonpathogenic infections at these early stages of infection, that have the potential to drive these different outcomes [46,47,48]. Additionally, studies in natural hosts have also established the key role of the immune activation and inflammation for the progression to AIDS and the development of comorbidities [49,50,51,52,53].

### Fifty Ways to Infect a Monkey—SIV/SHIV Strains for Use in Nonhuman Primates

**Figure 2:**
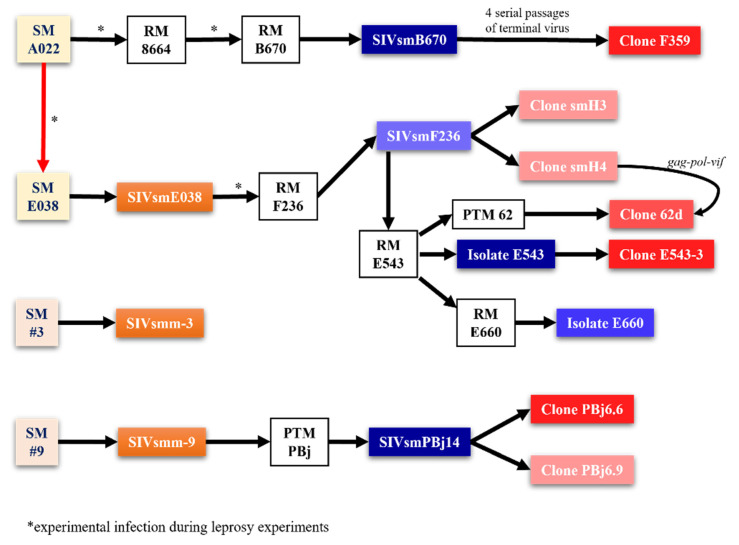
Isolation of various SIV strains used for NHP models. Different SIV strains vary in their pathogenic features, allowing for different uses by strain and animal species. The origin of different pathogenic isolates from sooty mangabeys at the Tulane National Primate Research Center (TNPRC) and those resulting from leprosy experiments, as designated by asterisk, from a sooty mangabey originally housed at the Gulf South Research Institute (now New Iberia Primate Center). Yellow and tan boxes are progenitor animals; white boxes are animals that were received serial passaging; orange boxes are initial strains isolated from SMs; blue boxes are strains isolated from serial passaging; red boxes are clones; darker tint indicates more prevalence in research.

While virtually every SIV strain can be used for studies in NHPs, there are several reference SIV strains that have been extensively used for studies in NHPs. In addition to the SIVmac lineage strains and the other strains accidentally generated through the kuru experiments carried out at the CNPRC (SIVmne, SIVstm and SIVmfa), many other SIV strains have been generated and employed over the years for experiments in macaques. Virtually concomitantly with the discovery of the SIVmac at the CNPRC, SIVsmB670 was isolated from macaques at the Tulane National Primate Research Center (TNPRC) [54]. There, in 1979, a female SM from the Gulf South Research Institute (currently New Iberia Primate Center) suspected of having leprosy was used in an extensive experiment involving serial passages of blood and tissues, with the goal of developing an NHP model for leprosy. Due to the very long incubation of leprosy, these experiments were only partially successful. Nevertheless, the passage of *M. leprae* to other SMs and RMs resulted in cases of full-blown AIDS in several macaques (particularly in the macaque B670) (Figure 2).

With each serial passage, the number of AIDS cases increased in the macaque groups and an SIVmac-related, albeit different, SIV could be isolated from both the RMs and SMs (Figure 2) [11].

After the discovery of SIVsmm in the SMs-naturally infected at the TNPRC, a new experiment aimed at rederiving a “clean” viral inoculum for the infection of the RMs was performed. Blood from the SM A022 was passaged into an uninfected SMM (E038), which was used as a source of virus for the infection of a RM (F236) [55]. The isolate SIVsmmF236 was lambda cloned into two relatively low pathogenicity clones (SIVsmH3 and SIVsmH4) [56]. Meanwhile SIVsmmF236 was passaged into a pigtailed macaque (PTM62) and a RM (E543). The isolate SIVsmmE543 was cloned into a highly pathogenic clone (SIVsmmE543-3) [57] and passaged into another naïve macaque (RM E660) [58]. SIVsmmE660 is currently a reference strain. It has a relatively high pathogenicity [59], and it is a tier 2 strain with regards to the neutralization sensitivity [60,61].

One of the issues with the SIVsmm family of reference strains is that they are susceptible to the TRIM5α restriction, unlike SIVmac-derived viruses, resulting in a wide range of viral loads (VLs) based on the TRIM5α genotypes [62]. Conversely, the SIVmac group accumulated mutations that conferred resistance to TRIM5a restriction [62].

More recently, both SIVmac and SIVsmm founder-transmitted infectious molecular clones have been derived for use in vaccine studies [63]. Meanwhile, for the purpose of cure studies, in which reservoir diversity and virus reactivation have to be investigated and which require viral diversity, both tagged [64,65] and barcoded [66,67] SIVmac clones have been produced, that combine the advantages of both infectious molecular clones (IMCs) (uniformity of the pathogenicity of the vial inoculum) and of the viral swarms, thus allowing a proper tracking of the number of viral variants that are reactivated during therapeutic interventions aimed at curbing the reservoir or analytical treatment interruptions (ATIs).

Finally, during a survey of SIVsmm diversity in the Primate Centers in the US, we identified multiple SIVsmm lineages that roughly mirror HIV-1 diversity and selected new potential references strains representative of every lineage [22]. For the vast majority of these new strains transmitted founder IMCs were derived and available.

For the studies of SIV pathogenicity in the African NHP natural hosts, several isolates have been used over the last few decades. Due to the endangered nature of most of the African NHP species, the vast majority of experimental infections in natural hosts are carried in African green monkeys (AGMs). Of these, the sabaeus monkeys are the model of choice due to the availability of a large wild population in the Caribbean. The reference strain for the studies in the sabaeus monkeys is SIVsab92018, which was derived from a chronically infected sabaeus monkey from Senegal [68]. Plasma from this animal was directly inoculated into naïve monkeys and collected during the acute infection for further use, without in vitro passage. A transmitted-founder clone has been derived from the acute plasma [69]. Additionally, SIVsab92018 was also directly passaged into pigtailed macaques and established a model of increased comorbidity prevalence and faster rate of progression while still recapitulating the pathogenic features of HIV infection [70]. Unlike in pigtailed macaques, direct passaging of SIVsab92018 (SIVsab) into RMs results in a state of functional cure, whereby the RMs naturally control the virus replication to below the limits of detection, immune populations are restored during chronic infection, and immune activation and inflammation (IA/INFL) are controlled back to baseline levels [71,72]. We utilize this model for experimental agent testing due to the ability to reactivate virus and bolster viral production by de novo infection off of antiretrovirals, thereby increasing resolution of viral reactivation.

Simian-human immunodeficiency viruses (SHIVs) are chimeric SIV-HIV viruses, which are developed as a method to try and mimic HIV-1 infection as best as possible in NHP models, with a large emphasis on vaccine development with the inclusion of the HIV glycoprotein. This is necessary due to the host restriction factors in NHPs that prevent productive infection of HIV-1 and HIV-2 [73]. The first SHIV developed was in 1992 with an SIVmac239 backbone that had its *rev*, *tat*, and *env* genes replaced with HIV-1 *rev*, *tat*, *vpu*, and *env* [74]. Replication was lacking in the first SHIV in vivo and thus researchers replaced the *env* with a dual-tropic CCR5/CXCR4 HIV-1 *env*, generating SHIV-89.6. SHIV-89.6 replicated to high levels in vivo, but still lacked some pathogenic features, such as sustained CD4^+^ T-cell depletion [75]. Serial passaging and additional modifications created SHIV-KB9, which recapitulated many features [76]. However, a major problem was that this virus was primarily CXCR4-tropic and resulted in modified (quickened) disease progression and did not properly represent HIV infection [77,78]. Importantly, the CXCR4-tropic SHIVs were overly sensitive to neutralizing antibodies, thus diminishing their usefulness in vaccine studies [78,79]. Thus, CCR5-tropic SHIVs became the focus of developing SHIVs [80,81,82,83,84]. In fact, SHIV_SF162P3_ has had great success in vaccine and broadly neutralizing antibody studies [82,85,86]. Serial passage of a SHIV using HIV-1_Ada_ *env* resulted in SHIV_AD8_ and its derivatives, which have also been used in vaccine, antibody, and therapeutics studies with relative success [87,88,89]. Nonetheless, even the CCR5-tropic SHIVs are not necessarily ideal due to the use of *env* sequences from chronically infected PWH and their passaging in NHPs results in modified *env* sequences [82,84,90]. They are therefore not as clinically relevant for vaccine studies as transmitted founder (TF) viruses which have special characteristics that increase fitness, and importantly, will have the relevant Env for targeting in vaccine or antibody studies [91]. Thus, with the new knowledge it became imperative to design transmitted founder SHIVs that do not require passaging [79], such as in vivo competition [92] with rational design and specific residue modifications (Env residue 375) [93,94,95,96] to improve binding and subsequent replication. However, issues still occur with spontaneous control and incomplete CD4^+^ T-cell depletion [95,96], likely due to insufficient viral replication that then prevents efficient immune escape. This also leads to issues with vaccine and bNAb studies, as the viruses do not properly recapitulate the pathogenicity of HIV, thereby potentially providing exaggerated protection.

## 3. Everything Put Together Falls Apart. A Brief Introduction to HIV/SIV Pathogenesis

### 3.1. HIV/SIV Reservoir Is Rapidly Seeded after Transmission

Studies of SIV transmission to RMs allowed us to characterize HIV transmission in great detail, with the goal of identifying windows of opportunity to prevent infection. The mucosal barriers highly hinder infection at the entry site, thereby limiting the infection to a very small, transmitted founder population, which then establishes a productive, disseminating infection in lymphatic tissues [97,98]. In the vast majority of PWH, a single virus initiates systemic infection [99] and the same is true for intravaginal exposure of RMs to low doses of SIVs [44]. While both CXCR4 and CCR5-tropic viruses can be found in sperm and vaginal secretions [100,101,102], the viruses that initiate infection (which are baptized TF viruses) are exclusively CCR5-tropic [99]. TF viruses have a specific fitness due to a lower glycosylation [103,104] and less sensitivity to type I interferons, especially IFN-α [105,106].

During sexual transmission, the virus migrates across the epithelial barrier at the site of entry via M cell transepithelial transport [107,108], dendritic cells (DCs) [109,110], and microtears in the epithelial layer [111]. HIV is then exposed to the local immune cell populations, including lymphocytes and macrophages, and rapidly infects and spreads through the primary target cells: CCR5^+^ memory CD4^+^ T cells [112,113]. The virus undergoes rapid dissemination from the site of entry. Thus, two days after intravaginal inoculation, the virus could be detected in the draining and even in the distant lymph nodes, before becoming detectable in circulation (5 days post-inoculation) [109]. Intrarectal transmission results in an even more rapid viral spread throughout the body [114,115], with no window of opportunity for potential interventions being observed upon intrarectal challenge [116]. As such, the study of the early events of HIV/SIV transmission showed that the immune response to infection is a double-edged sword: it helps establish the transmission bottleneck and eliminate virus, but the cellular activation also contributes to infection by increasing the amount of target cells at the site of entry [117].

### 3.2. Immune Response during Acute Infection Drives Viral Set-Point

During the acute infection, a massive immune response is triggered in response to the viral infection, with two separate waves of cytokines and chemokines. First, IFN-α, IFN-γ, inducible protein 10 (IP-10), interleukin (IL)-12, IL-15, and monocyte chemoattractant protein 1 (MCP-1) all rapidly increase prior to the peak of viremia. This is followed by increases in TNF-α, IL-6, IL-8, IL-18, and IL-12p40 [118,119,120,121]. Production of these molecules is mostly by dendritic cells (DCs), with additional production by monocytes, natural killer (NK) cells, and even T cells. Activation of plasmacytoid DCs is mediated by toll-like receptor 7 (TLR7) after endocytosis of HIV [122]. Myeloid-derived DCs are also responsible for cross presentation of antigens [123] while plasmacytoid DCs produce IFN-α primes T cells [124]. NK cells are direct effectors that are activated during HIV infection and clear infected cells through cytolysis and degranulation. NK cell cytotoxicity and antibody-dependent cell-mediated cytotoxicity (ADCC) result from binding of killer immunoglobulin-like receptors (KIRs), CD16, and the NKG2 protein family [125]. Through degranulation perforin and granzymes are released and induce target cell apoptosis [126]. NK cells produce various cytokines and chemokines, such as IFN-γ and TNFα [127] to limit viral infection and spread and β-chemokines which inhibit HIV entry to CD4^+^ T cells [128]. The adaptive CD8^+^ T-cell immune response begins prior to the peak of viremia. CD8^+^ T cells recognize foreign antigens that are presented on the cell surface by major histocompatibility complex (MHC) class I, stimulating the release of perforin, granzymes, and Fas ligand, leading to target cell apoptosis. CD8^+^ T cells also release IFN-γ and TNF-α into the microenvironment [129]. CD8^+^ T cells proliferation peaks around 2 weeks after the viral peak, their activation status being inversely correlated with viral set point. This proves that the post-acute viral control occurs via CD8^+^ T cells [130]. The emergence of the cellular immune responses exerts pressures on the virus at the transition from acute-to-chronic infection, and mutations are selected in the viral genome for CD8^+^ T-cell escape, leading to a continuous chess game between the CD8^+^ T cells that can respond to the new epitopes and subsequent viral escape [131,132]. B cells are also activated during acute infection, generate plasmocytes and initiate antibody production. Initially, the antibody response to HIV is non-neutralizing and does not impact the plasma viremia [133]. However, the antibodies are enriched for IgG3, suggesting that they have not gone through affinity maturation and may be due to the rapid dysregulation of lymphoid tissues where the B cells would interact with T cells for maturation [134]. Indeed, CD4^+^ T cells are rapidly depleted during acute infection which has deleterious consequences for proper adaptive immune responses [135] due to their role in providing stimulatory cytokines. Beyond that, the elimination of CD4^+^ T cells helps drive the mucosal dysfunction discussed later and eventually will reduce to levels defining AIDS without treatment.

The viral set point, which occurs around 5–6 weeks post-infection, marks the passage to the chronic infection phase, when the immune system and HIV have reached a pseudo-equilibrium of steady-state viral replication, immune-mediated clearance, viral escape, and T cell adaptation. It is thus unsurprising that the levels of plasma VLs are predictive for the rate of disease progression to AIDS [136,137]. Unlike VLs, the immune activation continues to rise into chronic phase, at which point it eventually hits the immune activation set point, in which CD8^+^ T-cell activation parallels the rate of CD4^+^ T-cell loss [138]. The immune activation set-point was also negatively correlated with the viral set point. In conjunction with data demonstrating that immune activation rapidly decreases with ART, it is likely that VLs is one of the drivers of the immune activation set-point [139,140,141].

### 3.3. Control at Last—Antiretroviral Therapy for HIV

The first antiretroviral (ARV), zidovudine, was approved by the FDA in 1987. Tritherapy, associating nucleozide reverse-transcriptase (RT) inhibitors with either non-nucleoside RT inhibitors or with protease inhibitors was introduced in 1996 and spectacularly impacted the outcome of infection: it completely suppressed viral replication and boosted the CD4^+^ T-cell counts [142]. Current ARVs target most of the HIV life cycle: entry inhibitors, prevent virus penetration in the target cells, by blocking CCR5 or CXCR4; fusion inhibitors prevent entry; RT inhibitors (nucleoside and non-nucleoside, NRTI and NNRTI, respectively) abort reverse transcription; integrase inhibitors, or integrase strand transfer inhibitors (INSTI) prevent viral integration; protease inhibitors (PI) prevent virion maturation [143]. The current first line of therapy is two NRTIs and either an NNRTI or INSTI, although new data support a two-drug regimen of dolutegravir (INSTI) and lamivudine (NRTI) for initial treatment [144]. ART thus effectively inhibits viral replication and decreases plasma VLs in PWH. In fact, most individuals will achieve viral suppression below the limits of detection, 50 vRNA copies/mL of plasma, as long as they maintain their regimen, and as long as their VLs are undetectable, the paradigm (and media slogan) has become “Undetectable = Untransmittable; U = U” [145].

While ART decreases VLs, it reciprocally restores CD4^+^ T cell counts, although this is highly variable and dependent upon the stage of disease progression and degree of immunodeficiency at treatment initiation. Studies have shown that the earlier ART initiation the better prognosis, with much better although incomplete restoration of CD4^+^ T cells [146,147,148], including in the GALT [149]. Studies also show that unsatisfactory CD4^+^ T cell restoration is correlated with higher mortality [150]. ART administration also contributes to a partial control of the levels of inflammation and immune activation, but cannot restore them to pre-infection levels [151,152] and similar to CD4^+^ T cell restoration, late ART initiation results in a more limited control of immune activation [153]. With advances in ART and accessibility, ART has drastically increased the life expectancy of PWH. While in the 2000s’ the life expectancy of a 21-year old PWH was 38 years, by 2016 it had increased to 57 years, a nearly 20 year increase [154]. Thus, ART has changed HIV infection from a life-threatening condition to a manageable chronic disease. Yet, life expectancy is still below uninfected persons (64-year life expectancy for 21-year old) [154] and, as discussed later, ART is not curative, nor does it completely prevent AIDS-related comorbidities.

## 4. At the Zoo. Nonhuman Primate Models for HIV Pathogenesis

### 4.1. Similarities and Recapitulation of Specific Pathogenicities

Nonhuman primates (NHPs) are excellent models for the study of HIV-1 due to the variety of pathogenic outcomes that can be induced through various combinations of NHP species and SIV strains. Further, due to their size it is possible to take far more consistent sample volumes (blood and tissues) than with other models, e.g., humanized mice, and their use allows for extensive tissue sampling that would otherwise not be possible in humans. Meanwhile, NHPs are outbred and more genetically close to humans than any other model, which allow a more rigorous modeling in NHPs compared to other inbred species. Of the several NHP species that can be utilized, cure research primarily uses RMs infected with SIVmac (either the reference swarm SIVmac251 or the infectious molecular clone SIVmac239) as the reference model. Notably, the SIVsmm family is the only one to induce pathogenic infection to RMs upon direct cross-species transmission of the virus from the natural host (SMs), and SIVsmm infection yields a pathogenic diversity with a wide range of outcomes of the infection (due to a partial TRIM5α restriction) [62], unlike the SIVmac infection [155,156,157,158,159,160]. Therefore, the combination of RM and SIVmac strains is the gold standard for HIV modeling because of it reproduces all the major features of HIV-1 infection in a condensed time frame [31]: (i) integration into host cell genome with similar integration site preference [161,162,163]; (ii) conversion to latency in infected cells; (iii) infected cell distribution to mucosal sites, lymph nodes, and peripheral blood [164,165,166]; (iv) Depletion of memory CD4^+^ T cells from the mucosal and lymphoid sites [37,38,39,40]; (v) chronic immune activation and inflammation, associating gut dysfunction and microbial translocation [39,113,117,167,168]. Although other species/strain combinations are available, they provide targeted usefulness, such as pigtailed macaques infected with SIVsab, which produces a highly pathogenic infection that is perfectly suited for the study of HIV/SIV-associated comorbidities [51,169,170], but long-term chronic illness is not easily achieved due to the very high pathogenicity of this infection, in which about 40% of SIVsab-infected PTMs progress to AIDS within the 6 months following the SIV challenge [70]. Further, for the study of HIV/SIV effects on the central nervous system (CNS), pigtailed macaques coinfected with SIVDeltaB670 and SIV17E-Fr are used because they quickly progress to immunodeficiency that associates CNS pathologies. This model showed that upon SIV infection, the CNS reservoir is seeded as early as 4 days, and that the macrophages are the major target cells of the virus in the brain [171]. Recently, a new model of RMs infected with a new molecular clone, SIVsmE-CL757, was reported to reproduce the CNS events without the rapid disease induction seen in PTMs [172,173]. At the opposite spectrum of pathogenic diversity from the SIVmac infection, RM exposure to SIVsab leads to a very robust acute SIV infection followed by a spontaneous complete viral suppression below the limits of detection, allowing for investigation of virus reactivation from the latency without the use of ART [72]. This is particularly helpful for understanding the reactivation potential of “shock and kill” latency reversing agents (LRAs), as the lack of ART enables de novo infection and therefore, larger viral bursts, allowing for easier detection of viral reactivation after the administration of latency reversal agents. The caveat is the inability to properly compare the reservoir before and after therapy due to the wide spread of the reactivated virus in the absence of ART.

### 4.2. Everything about It Is Inflammation and Immune Activation

Chronic T-cell immune activation and systemic inflammation are key pathogenic features of HIV/SIV infection [174,175]. T-cell immune activation and inflammation increase in response to virus early during infection, but they are not resolved after establishment of the viral setpoint, nor after viral suppression with ART [151,152]. In fact, the immune activation set point is one of the strongest predictors of disease progression [138,174,176], better than plasma VLs or CD4^+^ T-cell counts. This is due to the close association of the immune activation and inflammation with non-AIDS comorbidities and mortality in PWH and SIV-infected NHPs [49,50,51,52,53]. The determinants of chronic immune activation and inflammation in HIV/SIV infection are complex and multiple: (i) activation of the immune response through viral production and replication [139,140,141]; (ii) loss of gastrointestinal tract mucosal barrier integrity through the depletion of Th17 cells, which maintain mucosal barrier integrity [177,178]; (iii) microbial translocation from the lumen into systemic circulation and organs results from the damage to the mucosal barrier and epithelial tight junctions [179,180,181,182]; (iv) coinfections (e.g., hepatitis C virus [183], hepatitis B virus [184], herpes simplex virus type 2 [185], cytomegalovirus [186,187], and Epstein–Barr virus [188]) contribute to antigen-specific immune activation or pattern recognition receptor (PRR) activation and are increasingly active with progressive immunodeficiency [189]; (v) Toxicity of ART and other risk factors [52,190].

The chronic immune activation and inflammation impact disease progression through multiple pathways: (i) activated T cells become HIV/SIV target cells through expressing higher levels of coreceptors CCR5 and CXCR4 [191,192]; (ii) activation of NF-κB results in virus production [193]; (iii) constant activation results in increased T cell turnover and homeostatic proliferation, thereby decreasing the progenitor pool and inducing immune senescence [194,195]; (iv) increased expression of immune checkpoint expression (e.g., PD-1 [196,197,198,199] and CTLA-4 [200]) which results in decreased functionality (T-cell exhaustion) [201]; (v) collagen deposition and fibrosis (via transforming growth factor beta [TGF-β]) damages the fibroblastic reticular cell network in lymph nodes, resulting in aberrant immune reconstitution [202,203,204,205]; (vi) prolonged inflammation facilitates an increased risk of cancers [206,207]; and (vii) chronic inflammation damages vasculature and induces hypercoagulability, resulting in increased risk for cardiovascular diseases (CVD) [49,51,208,209,210]. In the end, these consequences result in both a higher frequency and earlier onset [4,5] of AIDS and non-AIDS comorbidities [211]. Comorbidities also include premature aging [211], sarcopenia [212], nonalcoholic fatty liver disease (NAFLD) [213], and HIV-associated neurocognitive disorder (HAND) [214].

Although there are several mechanisms that contribute to the chronic immune activation and inflammation, gut dysfunction and microbial translocation are arguably the largest contributors. Importantly, the onset of microbial translocation results in a vicious cycle of inflammation, mucosal barrier damage, and more microbial translocation; rinse and repeat [179,215]. Translocated microbial products activate monocytes and macrophages that then produce inflammatory cytokines (IFN-α, TNF-α, IL-1, IL-6, and IL-18), further activating the immune system [170,216,217]. This not only results in chronic immune activation and inflammation, but also drives HIV enteropathy, which was described in the earliest stages of the pandemic, when diarrhea, weight loss, malnutrition, malabsorption and villous atrophy were frequently diagnosed in AIDS patients [218].

### 4.3. Gut Dysfunction and Microbial Translocation Potentiate Immune Activation and Inflammation

HIV-associated GI pathology is triggered by the early and massive HIV-1 replication, and is characterized by immunological and structural abnormalities, including alterations of both the adaptive and innate mucosal immunity and substantial disruptions of the epithelial barrier [218,219,220]. These changes lead to increased local inflammation, microbial translocation and dysbiosis, and consequently to generalized immune activation and inflammation, and comorbidities [52]. This current pathogenic paradigm of AIDS, for which the impact of HIV infection on gut mucosa is the quintessential determinant of HIV infection pathogenesis, was made possible only through extensive use of NHPs. The animal models allowed invasive serial studies of the gut [219,221], and, as such, the reports on massive rapid depletion of the mucosal CD4^+^ T cells in NHPs preceded similar observations in humans by a decade [37,38,39,40]. Detailed comparative studies facilitated by invasive sampling at key time points of infection in multiple NHP models with different outcomes of SIV infection furthered this major paradigm shift in AIDS pathogenesis [222].

Intestinal mucosal lesions occur early in HIV infection and are rapidly established as part of a vicious circle in which gut damage, microbial translocation and IA/INFL potentiate each other [219]. Virus suppression with ART improves infection outcome, but frequently does not reverse GI dysfunction [52]. As a result, even in study participants in which the virus is suppressed for prolonged periods of time (some PWH received ART for >20 years), residual levels of IA/INFL nonetheless persist, leading to an only partial immune restoration at the mucosal sites, and an increased frequency accelerated aging and HIV-related comorbidities than in the general population.

Two major mechanisms are responsible for the gut dysfunction observed in HIV infection: (i) First, mucosal CD4^+^ T cell loss [218,220], the hallmark of HIV/SIV infection [37,223,224,225,226]. The virus infects and kills activated memory and effector CCR5-expressing CD4^+^ T cells, the major CD4^+^ T cell subset at the mucosal sites, particularly in the lamina propria of the gut. CD4^+^ T cell killing occurs in a caspase-1-dependent manner, resulting in a highly inflammatory form of death known as pyroptosis, which drives gut barrier dysfunction through production of inflammatory cytokines [227,228]. Exposure to microbial products may also divert the mechanism of mucosal cell death toward apoptosis [227]. Increased inflammation induced by microbial products is probably also responsible, at least in part, for enhanced bystander lymphoid and epithelial cell death and gut damage [227]. Similar to HIV-1 infection, CD4^+^ T cell depletion occurs early in SIV-infected macaques, is substantial, and is one of the correlates of the clinical outcome [50,220,229]. Depletion of T-cell subsets that control mucosal defense and homeostasis by limiting bacterial penetration and epithelial barrier integrity and function (i.e., Th-17 and Th-22) has been correlated with the development of intestinal pathogenesis [177,178]. Loss of T helper cells may also facilitate proliferation of opportunistic bacteria and damage to the gut [230]. In support of the direct role played by the CD4^+^ T cell loss in the gut damage is the observation that in patients with idiopathic CD4 lymphopenia, plasma lipopolysaccharide (LPS) levels are elevated, indicating increased gut permeabilization [231]. (ii) The second mechanism responsible for the gut dysfunction in HIV/SIV infections is through the loss of gut epithelial integrity. In progressive HIV/SIV infection, the excessive gut inflammation induced by virus replication damages the gut epithelium, allowing microbial products to first penetrate the gut mucosa and then translocate into the general circulation [180,219]. Immune cells exposed to these microbial products are subsequently activated through different PRRs, such LPS binding to toll-like receptor 4 (TLR4) [179], and thus lead to further gut damage by either directly fueling virus replication or indirectly through the release of proinflammatory cytokines and excessive cell death [230,232]. Conversely, the natural hosts of SIVs, which do not have progressive infection, have low levels of LPS in the periphery, indicating a lack of microbial translocation throughout infection [179,233]. AGMs were found to rapidly activate and maintain regenerative mechanisms in the gut mucosal tissue, thereby counteracting the vicious cycle [46]. Indeed, intravenous administration of LPS to SIV-infected AGMs resulted in systemic inflammation uncharacteristic of the infection [51,234]. These data were further supported by direct mucosal damage of SIV-infected AGMs through administration of dextran sulphate with similar results: systemic inflammation, T-cell activation, and increased plasma viremia [168]. Conversely, PTMs were treated with sevelamer, which binds LPS, and transiently reduced immune activation, inflammation, and even plasma viremia in the animals [182]. Thus, mucosal barrier damage, microbial translocation, and inflammation/immune activation are irrefutably intertwined.

### 4.4. Under African Skies—Study of Natural Hosts Demonstrates Important Differences between Pathogenic and Nonpathogenic Infections

The natural reservoir of SIVs is represented by African NHPs. Over 40 species of monkeys in Africa are infected with species-specific SIVs [18]. In their natural hosts, such as AGMs, SMs and mandrills (MNDs), SIV infection appears to be nonpathogenic [18,235,236]. In these species, disease progression is highly uncommon, only occurring in a handful of animals which had greatly outlived their normal life expectancy [237,238].

Extensive studies performed over the last three decades, allowed us to thoroughly characterize the pathogenesis of SIV infections in their natural hosts. Through these comparative pathogenesis studies, we identified similarities and differences between the pathogenic and the nonpathogenic infections, thus establishing features that were specifically associated with the progression to AIDS in the pathogenic infections [239]. The most important shared feature of the pathogenic and nonpathogenic HIV/SIV infections is the robust acute viral replication, followed by high steady-state replication that is higher than in the majority of untreated chronically PWH [68,160,240,241,242,243,244,245]. Meanwhile, African natural hosts similarly undergo a severe CD4^+^ T-cell depletion at the mucosal sites with the same order of magnitude as that observed in PWH and pathogenic SIV infections, in line with the primary target cell of SIV in African NHPs being the CD4^+^ T cell [37,39,113,117,233,246,247,248]. Furthermore, the humoral and cellular immune responses are similar between the pathogenic and nonpathogenic SIV infections [13,17,239,249,250,251].

These common features between pathogenic and nonpathogenic infections suggest that the lack of disease progression in natural hosts is not the result of a viral attenuation. Indeed, the rare cases of AIDS documented in African NHPs [237,238] and the observation that direct SIV cross-species transmission from their natural hosts to macaques results in pathogenic infections that progresses to AIDS [70,160,252] confirm that control of disease progression is independent of the virus and instead relies on host adaptations. This likely occurred because of the SIV-African NHP host coevolution occurring over hundreds of millennia [16,22,253,254,255]. This virus-host coevolution allows natural hosts to counteract the deleterious consequences of the SIV infection and resulted in phenotypic features of natural hosts that contribute to the prevention of disease progression to AIDS [16,253,254,256,257,258,259,260,261]. In particular, these would be: few target cells (CCR5^+^ CD4^+^ T cells) at mucosal sites [22,247,262,263] and downregulation of CD4 on helper T cells when they transition to memory phenotype [262,264]. The usage of CXCR6 as a coreceptor may also server to further preserve CD4^+^ cells in AGMs and sooty mangabeys [261,265]. Furthermore, NK cells are found at much higher levels in the lymph node follicles of AGMs than in pathogenic models [266], while also displaying a greater number of terminally differentiated NK cells and increased SIV-specific activity [267]. Thus, this mechanism can help explain the reduced damage occurring in nonpathogenic infection.

The main factor behind the lack of disease progression in the natural hosts of SIVs is their ability to actively control chronic immune activation and inflammation [47,239], the main drivers of disease progression and mortality in PWH [174,175]. Chronic systemic T-cell immune activation and inflammation are kept at bay through an exquisite ability of the natural hosts of SIVs to maintain the integrity of the mucosal barrier throughout the course of SIV infection [48,168], due to specific healing mechanisms recently described [46]. This lack of mucosal dysfunction allows the natural hosts to avert microbial translocation [179,233], in stark contrast to the pathogenic HIV/SIV infections, in which microbial translocation occurs as a result of acute viral replication and proinflammatory responses causing extensive damage to the intestinal mucosa [268].

### 4.5. How the Heart Approaches What It Yearns—SIV as Models for the Study of HIV-Related Comorbidities

Although ART is able to curb viremia, there is still a disproportionate risk of non-AIDS comorbidities in PWH, with higher rates of CVD, kidney disease, hepatic disease, and other events [269], replacing opportunistic infections as the leading causes of mortality and morbidity. In fact, from 2000 to 2010, AIDS-related deaths in a French study group decreased from 47% to 25% [270], while a multicohort study showed a decrease from 34% to 22% in 1999–2000 to 2009–2011, respectively [271]. The transition from AIDS-related mortality and morbidity to non-AIDS is associated with an increased lifespan for PWH, yet there is still a life expectancy deficit, averaging 8 years less [151,272]. Further, as the PWH population ages, there is an increasing risk of multiple comorbidities arising per individual than in the uninfected population [273].

Due to the differences in natural hosts and pathogenic infections, a method to increasing our understanding of HIV pathogenesis is to compare the two and find differences in host biology. This strategy has allowed for incredible progress in our understanding of HIV transmission, pathogenesis, prevention, and treatment [31,53,274,275]. SIVsab, the SIV that naturally infects AGMs, also infects PTMs. Both infections present with high VLs, but completely opposite disease outcomes. SIVsab-infected AGMs do not progress to AIDS, while SIVsab-infected PTMs present with nearly all pathogenic features of HIV infection and readily progress to simian AIDS [53,169]. As mentioned earlier, comparisons between the two models were integral to understanding immune activation, inflammation, gut dysfunction, and microbial translocation in HIV infection. Indeed, other comorbidities are also investigated with NHP models. PWH are at an undeniably higher risk for CVD [276], which is recapitulated in both SIVsab-infected PTMs and SIVmac-infected RMs. These models present with hypercoagulation, demonstrated by significant increases in D-dimer and thrombin-antithrombin complex. This is especially prevalent in the SIVsab-infected PTMs, where these biomarkers were increased early after infection and associated with cardiovascular lesions and were greatly indicative of progression to AIDS and mortality [51]. Additionally, thrombotic microangiopathy was present in multiple organs, while myocardial hypertrophy, fibrosis, myocarditis and infarction were also observed [51]. This model has also shown that therapeutic interventions for reducing microbial translocation, immune activation, and inflammation resulted in decreased hypercoagulation, further supporting the role of immune activation and inflammation in hypercoagulation [169,182].

Liver dysfunction is frequent in PWH and has multiple sources: (i) infection of the Kupffer and stellate cells in the liver [277,278,279]; (ii) microbial translocation and the chronic inflammation [278,280]; (iii) coagulopathy [52,281]; (iv) cofactors, e.g., hepatitis C virus coinfection [282] and excessive alcohol consumption [283]; and (v) ART toxicity [280]. SIVsab-infected PTMs demonstrated inflammatory infiltrates and hepatic fibrosis, which together resemble chronic active hepatitis [53], and RMs demonstrated that the liver is highly involved in clearing virus from circulation [284]. Further, CD4^+^ T cells are greatly reduced and CD8^+^ T cells are highly increased in the liver after SIV infection, indicating the liver as a site of antigenic stimulation and CD4^+^ T cell depletion [285,286].

Respiratory comorbidities are on the rise with PWH living longer, such as chronic obstructive pulmonary disease (COPD) [287], however the mechanisms are not well elucidated. The SIVsab PTM model was also used to investigate pulmonary lesions that may play a role in the rise of COPD in PWH. In the PTMs, early infection presented with immune infiltrates in the lung parenchyma and near large bronchi. During chronic infection, emphysema and thickened alveolar walls are observed with disruption of the lung architecture and fibrosis, in direct contrast the SIVsab-infected AGMs which presented with no immune infiltration or subsequent lung disruption [53]. The elimination of interstitial macrophages present in the lungs is also a cause for pulmonary disease [288].

Acute renal failure and chronic kidney disease are associated with advanced immunodeficiency and age, therefore greatly increasing the risk in older PWH [289]. HIV-associated nephropathy (HIVAN) can quickly progress to end-stage renal disease and mortality if left untreated [290]. However, like respiratory comorbidities, the mechanisms are not fully known. Although several ART drugs have been associated with kidney damage, they do not explain the full extent of renal disease [291]. It is believed that chronic immune activation and inflammation are likely the main mechanism because early initiation of ART, which allows for better maintenance of immune function, minimizes the risk of kidney disease in PWH [291]. In RMs infected with SHIV_KU-1_, researchers found the equivalent of HIVAN with glomerulosclerosis and collapsing glomerulopathy [292], and another SHIV-infected RM presented with nephrotic syndrome: peripheral edema, hypoalbuminemia, and proteinuria [293]. In our model of SIVsab-infected PTMs, we have shown similar kidney pathologies to HIVAN, including hyperplasia of the Bowman capsule epithelial lining, glomerulosclerosis and collapsing glomerulopathy, and interstitial nephritis [53].

The rate of HAND in PWH has drastically decreased after the advent of ART, but less severe neurocognitive issues remain and risk increases with age [294]. HAND is a spectrum that includes asymptomatic neurocognitive impairment (ANI), mild neurocognitive disorder (MND), and HIV-associated dementia (HAD), with HAD being the most severe form. The spectrum is defined by neuropsychological testing and functional status assessments. The biomarkers accessible by blood for HAND are not very specific: CD4^+^ T cell count at nadir of depletion, sCD14, sCD163, and viral DNA, all of which can be associated with general progression [294]. Cerebrospinal fluid, however, shows associations with neuronal injury markers, as well as inflammation, demonstrating more specific markers [294]. Further, neuroimaging markers are helpful and functional MRI has demonstrated accelerated aging in the brains of PWH [295]. Animal models allow for invasive approaches and euthanasia further permits investigation into brain pathologies at necropsy with SIV-infected PTMs being the model of choice [296,297].

## 5. For the Cure, Whenever We May Find Her

### 5.1. The Need for an HIV Cure

An essential step of the HIV replication cycle is integration into the host genome, whereby it can use host cell machinery to produce its viral mRNA products and RNA genome. Once the viral latency is established, the cells cease to produce viral products, and they can no longer be recognized by the immune system, allowing the provirus to persist in these cells indefinitely [298]. The totality of the integrated proviruses forms the latent reservoir; the HIV-infected CD4^+^ cells that contain integrated HIV and revert to a resting state with altered gene expression, for example reduced NF-κB, which is normally triggered by T cell activation, results in a pool of hidden, activatable provirus [299]. While ART effectively suppresses the circulating virus [300], the reservoir cells are not impacted by ART, and treatment cessation is always followed by a viral rebound with VL levels similar to those observed pretherapy [301,302,303,304]. The source of this virus rebound is the latent reservoir, which can be reactivated by multiple stimuli inducing T-cell activation and latency reversal. This is the scientific basis of the need for a life-long adherence to ART. ART was one of the greatest achievements of modern medicine, yet long-term toxicity, viral resistance, stigma, and costs, all call for an effective HIV cure aimed at complete HIV eradication from PWH. ART does not completely restore the immune system, nor eradicate HIV. Multiple strategies towards an HIV cure are pursued [305,306,307,308,309,310,311,312,313,314,315,316,317,318,319,320,321,322,323], but none effectively curbed the reservoir nor induced robust and durable virus control, except the hematopoietic stem-cell transplantation, which is not scalable and has unreasonable limitations [7,8,324,325]. The major barriers to a successful HIV eradication are: (i) HIV persistence in latently infected cells invisible to immune responses; (ii) inability of a damaged/exhausted immune system to eliminate HIV-infected cells; and (iii) chronic INFL that persists on ART [326,327,328,329,330].

### 5.2. The Latent Reservoir Currently Prevents HIV Cure

The existence of the HIV latent reservoir is the primary obstacle for cure HIV eradication from the host. The latent reservoir is established immediately following infection, as early as 3 dpi, and prior to detectable viremia [28,29,30,331], in resting CD4^+^ T cells: [303,304,332,333,334,335] with different immunophenotypes: central memory [312,335,336], transitional memory [312,336], stem cell memory T cells [337], Tregs [338], and follicular T helper CD4^+^ cells [339]. In addition to the CD4^+^ T cells, macrophages and monocytes can be latently infected by HIV/SIV [340]. Dendritic cells are suspected to contribute to the reservoir by carrying SIV/HIV virions on their surface [341]. Latently infected cells lack a specific surface marker which would allow specific targeting of the latently infected cells [342] which is one of the major barriers against an HIV cure.

The prospect for an HIV cure became a reality after the success of the “Berlin patient”, a PWH who underwent allogeneic bone marrow stem-cell transplantation to treat acute myeloid leukemia. The donor was chosen specifically for homozygosity for the *CCR5* Δ*32* allele, and thus without a functional CCR5 coreceptor and resistance to HIV infection; as a result, after two stem cell transplantations, graft-versus-host disease, irradiation, immunosuppressive therapies and whole body irradiation, the Berlin patient presented with a drug-free HIV remission [343] which lasted for 12 years prior to his death. A second patient that underwent a similar procedure with a *CCR5* Δ*32* allele donor (the “London patient”) is also reported to be in remission [8]. Yet, while cure research got a tremendous boost leading to major improvements in our understanding of the nature of viral reservoirs and of the mechanisms of HIV latency in the decade following this remarkable success story, this procedure is not scalable, and, as such, there were not many subsequent cases of success in this field. The “Boston patients,” which also went through a similar transplantation (yet with stem cells from donors with intact *CCR5*), rebounded by months 3 and 8 post-ART interruption [344]. As such, these cases demonstrated that standard bone marrow transplantation is not sufficient to cure HIV. Furthermore, while ART can suppress plasma viral RNA to below limits of quantification, cessation of ART results in viral rebound in virtually every situation, including the “Mississippi baby,” who was on ART from 30 h to 18 months of age, and was thought to be functionally cured [345]. In this patient, the virus eventually rebounded 2 years after interruption of ART [346], due to the persistence in the latent reservoir. Additional cases of people believed to have been cured or functionally cured post-cessation of treatment based on conventional measurements of the viral reservoir include the VISCONTI cohort [347] and a South African child [348].

On the other hand, NHP models have demonstrated that early initiation of ART does not prevent the viral rebound post-therapy interruption [28], indicating that the reservoir is established very early in infection, suggesting that interventions aimed at curing HIV infection will need to curb the reservoir rather than prevent its formation. Nevertheless, in the same NHP studies, a delay in virus rebound at the cessation of art was observed in macaques in which therapy was initiated very early, at 3 dpi, prior to detectable viremia. In a case of an PWH treated with allogeneic stem cell transplantation for treatment of acute lymphoblastic leukemia, researchers found that the virus rebounding nearly one year after treatment interruption was phylogenetically distinct from the HIV strain detected in PBMCs prior to transplantation [349]. These rebounds illustrate that not only we do not have an effective cure strategy, but we also have not fully mastered the diagnostic tools necessary for monitoring the effectiveness of various cure strategies, indicating a need for more effective methods and strategies.

### 5.3. Multi-Trick Pony—Mechanisms of HIV Latency Establishment

HIV latency was first described with in vitro experiments demonstrating that cells that survived infection did not produce virus, but could be induced with 5-iodo-2′-deoxyuridine [350]. Shortly after, studies showed the stimulation of HIV transcription was regulated by the same pathways that induce T-cell activation [351,352,353], which suggested that activated CD4^+^ T cells were not likely to support latency. However, resting CD4^+^ T cells poorly support productive infection [354,355]. Thus, the paradigm of reservoir formation became the transition of infected, active CD4^+^ T cells to a resting state, and it was proven in 1995 that resting CD4^+^ T cells from PWH can harbor replication competent provirus [356]. In fact, multiple in vitro studies have since supported that infected, activated CD4^+^ T cells gradually transition back to the resting state and support latent infection [357,358,359,360,361,362,363].

The preferential integration of HIV into transcriptionally active sites [364,365,366] suggests that HIV expression is at least partially independent of the host gene expression. After integration, two nucleosome structures, Nuc-0 and Nuc-1, are formed at the 5′ long terminal repeats (LTR), blocking transcription initiation by RNA polymerase II (RNAP II) [367]. These nucleosomes are associated with epigenetic modifications that contribute to HIV latency: histone deacetylation [368,369,370] and methylation [371,372,373], leading to contraction of the chromatin structure and repression of transcription. Further, not only are the modifications observed, but the histone methyltransferases and deacetylases are also associated with the LTR [368,371,373,374] and recruitment is facilitated by various transacting factors [361,375,376,377]. These data also help explain the strong reactivation potentials of various HDAC inhibitors.

Transcriptional interference is another mechanism driving HIV latency, depending on the relative orientation of the provirus in the host gene. With same sense polarity, the tendency to integrate into active sites can readily cause elongation of the host gene to displace transcription factors at the HIV LTR, thereby preventing transcription initiation [378,379]. When the provirus is integrated in the opposite polarity to the host gene, transcriptional interference manifests with collisions between the elongation complexes of the host gene and HIV transcription [380].

Recruitment of the host factor positive transcription elongation factor b (P-TEFb) from the 7SK small nuclear ribonucleoprotein (snRNP) complex is facilitated by competitive binding of HIV Tat (transactivator of transcription) to HEXIM1, causing the release of P-TEFb [381,382]. P-TEFb then mediates the phosphorylation of RNAP II [383,384] and Spt5 [385], preventing early termination of transcription, which leads to efficient transcription elongation. The bromodomain proteins BRD2 and BRD4 act competitively with HIV Tat for P-TEFb binding, resulting in diminished transcription elongation [386,387]. Thus, it is not surprising that BRD2/4 binding by bromodomain inhibitor JQ1 results in viral reactivation [388,389].

### 5.4. Reservoir Decay Is Not Curative

Early reservoir decay modeling suggested that maintaining ART for 7.7 years may be able to completely eradicate the latent reservoir [390], yet this has been clearly debunked, with PWH reaching decades without complete clearance on ART. Newer modeling from PWH on ART indicates that the half-life of total HIV DNA is 42 years, whereas the intact provirus half-life is 7 years [391], thereby negating the theory of eradicating HIV-infected cells solely through sustained ART. The data demonstrate that early ART initiation is beneficial for the rate of reservoir decay [391], but still not enough to eliminate the reservoir.

### 5.5. Somewhere Researchers Cannot Find Me: Technical Obstacles towards Reservoir Quantification

A technical obstacle towards the eradication of the latent reservoir is the lack of a proper quantification of the inducible virus. Initial measurements used cell-associated HIV DNA (caDNA) to quantify the latent reservoir [301,356,392], but it soon became clear that only a fraction of these cells were capable of producing infectious virus [332], thus demonstrating an inherent issue with measuring caDNA: not all cells may be relevant for the recrudescence of infection after ART cessation. Full genome sequencing revealed that the proviruses forming the latent reservoir are both intact and defective [393], further diminishing the significance of caDNA as a measurement of the inducible virus. Reservoir quantification took a step further when a the quantitative viral outgrowth assay (qVOA) was established to be the gold standard for measuring the inducible virus [332]. The qVOA dilutes purified, resting CD4^+^ T cells from HIV donors and activates them with a stimulant (e.g., phytohemagglutinin [PHA] [332], phorbol 12-myristate 13-acetate [PMA] and ionomycin [394], or anti-CD3/CD28 [303,395]) in the presence of feeder cells (irradiated PBMCs). The original method of activation (PHA) was shown to induce activation in nearly all resting T cells [396], and minimal differences were seen with the other activation methodologies [397]. However, qVOA is time consuming because it requires that stimulated cells are cultured for 14–21 days so that enough p24 can be generated for quantification via ELISA [335]. An alternative method of using PCR as the end quantification [398] decreases time consumption, but has its own issue of viral RNA being produced from a fraction of defective proviruses, thus artificially increasing the size of the replication competent reservoir [399].

An additional problem with the qVOA is that the in vitro stimulation lacked efficacy in reactivating all of the replication-competent viruses from the purified CD4^+^ T cells, as demonstrated by the observation that multiple rounds of cell activation yielded additional virus [400,401] and sequencing with subsequent infection of cells in vitro confirmed replication capabilities of wells negative for viral outgrowth [400]. Beyond the immediate ramifications towards quantification, this also pointed towards another barrier of HIV cure: HIV-infected T cells can activate and clonally expand without reactivating virus, thereby avoiding the immune response while bolstering the reservoir size [402]. A way to mitigate the problem of incomplete activation, is to perform sequencing for determining the percentage of provirus that has intact provirus.

For full-genome sequencing, researchers extract genomic DNA and use nested PCR with limiting dilutions. The PCR products are then run on agarose gels and extracted for sequencing [403], thus this technique minimizes errors, but it also is highly time consuming and intensive. Unfortunately, reducing the time constraints and labor by using subgenomic sequencing introduces detection and accuracy problems due to either defects in regions outside of the amplified region or deletions overlapping the amplified region [404]. Utilizing next-generation sequencing is one method to increase efficiency and cost effectiveness [405,406,407] and has higher sensitivity than Sanger sequencing, allowing for a better detection of mutations [408]. The recently developed intact proviral DNA assay (IPDA) is based on digital droplet PCR (ddPCR) multiplex technology [409]. It uses primers against conserved regions of *env*, the packaging signal (PS) and Rev-response element (RRE), to elucidate defective versus intact provirus. The benefit of this assay is that it requires few cells (5 million CD4^+^ T cells) and does not have the inefficiency of long-distance PCR. The caveat is that by only detecting a small region of the genome (~2%), the IPDA can easily miss other defects that would render the virus replication incompetent [409] and also has issues with polymorphisms affecting detection [410]. To mitigate this issue, a combination of quadruplex qPCR and NGS, termed Q4PCR was developed. Like IPDA, Q4PCR also uses the PS and RRE regions, but also includes primers for *pol* and *gag*. Using Q4PCR with NGS showed that IPDA had high variability in detection of true intact provirus due to missing polymorphisms outside of the amplified sequence [411]. Additional head-to-head comparisons between these two methods are warranted.

Nonetheless, the proviral sequencing demonstrated that only a small fraction (~5–7%) of the proviruses are intact, regardless of the timing of ART initiation [393] and accounts for around 60 per million CD4^+^ T cells [400], a 60-fold increase in the number of replication competent virus estimated by qVOA [412]. These data were initially promising for the eradication of HIV, as it suggested the possibility of eliminating far less infected cells than previously thought. However, studies demonstrated that the ability of defective proviruses to produce viral proteins may be stimulating the immune system, thus contributing to the viral pathogenesis [399,413,414].

### 5.6. SIVmac-Infected RMs as a Model for Cure Research

In addition to the general roadblocks to cure, there are specific limitations to cure research in humans [31,415]: (a) ART cannot be stopped without the risk of emergence of drug-resistant strains; (b) residual viral replication prevents proper characterization of the reservoir; and (c) invasive sampling of multiple potential reservoir sites is limited. These limitations make use of animal models imperative for the study of the viral reservoir and for testing cure strategies. Although humanized mice have potential for cure research [416,417,418,419,420], size limitations of individual animals prevent detailed reservoir assessment. Therefore, the model of choice is the SIVmac-infected RM on ART.

HIV and SIV share key features of virus persistence: (a) HIV/SIV DNA are similarly integrated in the target cell genome [421,422,423]; (b) response to interferons results in transcriptional control of long terminal repeat sequences (LTRs) through histone acetylation favoring HIV/SIV DNA persistence [424]; (c) costimulatory signals induce latent HIV/SIV without co-engagement of T cell receptors [425]; and (d) distribution of cells containing HIV/SIV DNA and RNA sequences in blood, LNs, and mucosal sites are similar in humans and RMs [164,165,166]. SIVmac infection of RMs reproduces all the stages of HIV infection in a shorter time frame. These characteristics demonstrate similar reservoir dynamics between HIV and SIV infection. Historically, SIVmac was difficult to control with ART, requiring complex and expensive drug combinations [426]. Emergence of new integrase inhibitors and use of coformulated drugs now allow SIVmac suppression with ART regimens that are similar to, or the same as, those used in HIV infection [427], thus further establishing SIVmac-infected RMs as the gold standard model.

## 6. Still Searching after All These Years: Strategies towards an HIV/SIV Cure

**Figure 3:**
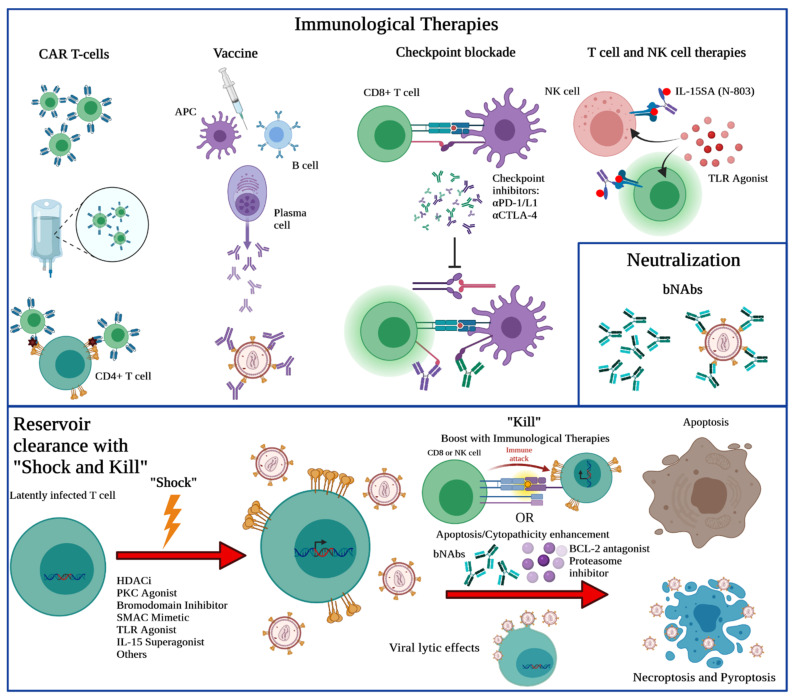
Cure strategies focusing upon clearance of the latent reservoir. There are many different therapeutics that are being investigated for HIV cure. CAR T-cells are engineered with chimeric receptors to better respond to virally infected cells and eliminate them. Vaccines can induce B cell production of antibodies against virus, but also T-cell responses to help clear the infected cells. PD-1/L1 and CTLA-4 are suppressors of T-cell activation and by blocking these interactions, there can be increased T-cell activation. Il-15 superagonist and TLR agonists can be administered to individuals to bolster the cell-mediated immune response. Broadly neutralizing antibodies (bNAbs) can be directly administered to neutralize virus. Of note, eradication of the latent HIV reservoir and establishment of potent immune responses may result in eventual functional or sterilizing cure and will likely require combination approaches as shown in this schematic with various potential drugs, small molecules, or interleukins as treatment.

Numerous strategies have been proposed for the elimination of the viral reservoir: (i) ART intensification [428,429,430,431]; (ii) “block and lock” permanent transcriptional silencing [307,432]; (iii) gene editing of CCR5 [433]; (iv) chimeric antigen receptor (CAR) T cells [322,434,435]; (v) apoptosis promotion and viral cytopathic effect enhancement [436,437]; (vi) bone marrow transplantation [344]; (vii) broadly neutralizing antibodies [438,439]; (viii) vaccines and therapeutic vaccines [440]; (ix) regulatory T cell (Treg) manipulation and depletion [338,441,442]; (x) use of checkpoint inhibitors to enhance HIV-specific immune responses [443,444,445,446]; and (xi) “shock and kill” (Figure 3). However, these have been met with limited success.

In spite of some promising results being reported by previous studies, none of the LRAs tested so far showed enough potency to justify their large-scale use as HIV cure agents [394,447,448]. In addition to this lack of efficacy resulting in insufficient reservoir reactivation [448], some LRAs were reported to induce a massive, indiscriminate T-cell activation that can be detrimental to the host health or even lethal [449,450]. Finally, some LRAs, particularly HDACi, were reported to have a negative impact on the cell-mediated immune response [451,452,453,454].

Other cure strategies also have issues with efficiency, be it the lack of reducing residual viremia or reservoir size with ART intensification [455], insufficient virus reactivation with Treg manipulation [441] and additional toxicity when combined with ART [442], lack of viral clearance in response to checkpoint inhibitors [443,444], inherent resistance and escape mutations against broadly neutralizing antibodies [456], lack of great enough efficiency and long-term stability of gene therapies and CAR T cells [322,457], or safety concerns of bone marrow transplants coupled with the lack of success [344,458]. As such, after more than a decade of intensive research, the end is still not in sight.

### 6.1. ART Intensification as a Cure Strategy

ART intensification was thought to potentially alleviate some of the residual virus replication in patients on ART [459,460]. However, several studies have demonstrated that ART intensification does not solve the issue of low-level residual replication. In fact, efavirenz, lopinavir/ritonavir, and atazanavir/ritonavir [428], raltegravir [461], dolutegravir [462], and maraviroc [463] were all used to intensify ART, to no avail. Due to the major role of the gut in HIV pathogenesis, another study focused upon the gut when attempting ART intensification with raltegravir and maraviroc. With this combination, there was no benefit to the immune populations of the gut, decreases in inflammatory markers beyond what was seen in the control group, further demonstrating the futility of ART intensification [464].

### 6.2. “Block and Lock”—Transcriptional Silencing for HIV Functional Cure

HIV transcription involves both viral and cellular machinery. During viral production, HIV initially transcribes short, completely spliced transcripts that create the Tat and the regulator of virion expression (Rev). Tat acts as an autoregulator for HIV and binds to the HIV transactivation response element (TAR) of the HIV promoter. This allows for the recruitment of the RNAP II elongation factor, P-TEFb, that results in transcription initiation and elongation [465,466]. One strategy for an HIV cure is to force the viruses into latency, by blocking viral transcription and locking the viral promoter into a late state, thereby preventing disease progression. This is called the “block-and-lock” strategy, and it utilizes antagonists to viral proteins or host transcription machinery [467]. There are several different targets of “block-and-lock”, but the most investigated is the use of Tat inhibitors, due to Tat’s role in HIV transcription [465,466]. NullBasic was the first Tat-inhibitor developed in 2009 and is comprised of a transdominant Tat mutant which is meant to outcompete wild-type Tat [468]. In vitro, NullBasic-expressing cells produced significantly fewer virus and reduced the efficacy of viral reactivation after PMA stimulation. However, this protein did not completely inhibit production of full-length mRNA and also had to be stably expressed in the cells to silence transcription [469]. Didehydro-cortistatin A (dCA) [470] is currently the most advanced small molecule inhibitor developed. In cell cultures, dCA was shown to inhibit viral activation and even inhibit reactivation after stimulation with prostratin [471]. When administered to HIV-infected, ART-treated, humanized bone marrow/liver/thymus (BLT) mice, treatment of dCA significantly decreased the aggregate number of viral RNA copies vs. controls and increased the time to viral rebound after analytic treatment interruption. There, all mice rebounded by 10 days post-interruption in the control group and 19 days post-interruption in the dCA group [471]. Unfortunately, other block-and-lock small molecule inhibitors, such as HSP90 inhibitors, Jak-STAT inhibitors, and kinase inhibitors, are more prone to side effects, due to their roles in host transcription [472]. In fact, characterization of CDK and the mammalian target of rampamycin (mTOR) inhibitors indicated that due to cellular toxicity, the vast majority these inhibitors had to be discarded [473]. This illustrates the difficulty of developing small molecular inhibitors against host proteins for HIV silencing.

Finally, transcriptional gene silencing was another method of silencing that different groups tested. It is based on the use of short hairpin RNAs and short interfering RNAs that are able to reduce viral burden, but run into issues with delivery methods [467].

### 6.3. Gene Therapy and Engineered CAR T Cells for HIV Cure

In vitro and ex vivo gene therapy with CRISPR/Cas9 has been able to disrupt proviruses [474,475,476], but the lack of a systemic delivery mechanism and of target effects in humans hinders gene therapy as an HIV intervention [477]. Nonetheless, advancements in gene therapy have allowed for engineered immune cells to be used to combat HIV. For instance, engineered T cells with CAR against HIV have shown promise in eliminating HIV, but are still hindered in vivo by lacking sustained activity of the cells for the time period necessary for eradication, off target effects, the threat of CAR immunogenicity, and the possibility of inducing a cytokine storm in the patients [322]. One of the large issues with CAR T cells is protection of the CAR T cells against HIV infection. Thus, researchers developed conjugated coreceptors, C34 conjugated to CCR5 or CXCR4, and tested them in vitro. These were found to be able to protect against HIV infection, and a conjugate of CXCR4 and 34 peptides from the heptad repeat domain 2 (HR2) of gp41 (C34) demonstrated better protection than the C34-CCR5 conjugate [478]. Following these results, new dual CD4-CAR T cells, expressing both 4-1BB/CD3-ζ and CD28/CD3-ζ ectodomains with a co-expressed C34-CXCR4 fusion inhibitor, to protect against HIV infection, were tested in humanized BLT mice. The results demonstrated elimination of infected cells in vivo, including memory CD4^+^ T cells, while also reducing the loss of CD4^+^ T cells during acute phase, and decreasing plasma viremia and cell-associated HIV DNA (from memory CD4^+^ T cells). However, the protection from HIV was eventually lost over time, demonstrating the need to develop chimeric cells with complete resistance to HIV [479].

### 6.4. Enhancing Apoptosis and Cytopathic Effects as a Cure Strategy

Due to the production of the HIV protease, infected cells are pushed to a pro-survival state. The HIV protease cleaves procaspase 8 to create Casp8p41, which is capable of binding to BAK and inducing apoptosis [480,481,482,483]. However, in cells producing high levels of the antiapoptotic protein BCL-2, such as central memory CD4^+^ T cells, which form the bulk of the HIV/SIV reservoir, Casp8p41 binds to BCL-2 instead of BAK, thereby neutralizing the proapoptotic ability of Casp8p41 and inducing the pro-survival phenotype. Transcriptional profiling of CD4^+^ T cells that survived coculture with HIV-specific CTLs demonstrated that BCL-2 is a major overexpressed marker, further substantiating its role in persistence. Venetoclax is a Bcl2 antagonist, which was shown to induce the death of HIV-infected cells in vitro, with a strong selectivity towards infected cells [436,437]. Another proapoptotic drug is ixazomib, a proteasome inhibitor. Because BCL-2-Casp8p41 complex together and become polyubiquinated and then degraded at the proteasome, the use of a proteasome inhibitor prevented degradation of Casp8p41, allowing for increased activity and binding to BAK. When administered to cells in vitro, ixazomib was able to increase death of HIV-infected cells with the added benefit of reactivating HIV via NF-κB [484]. It thus appeared that the combination of the two drugs is ideal for first increasing viral reactivation, then induce a proapoptotic state, while preventing the decay of the Casp8p41. When this combination was attempted in vitro, the reduction of HIV-infected cells surpassed that of either drug alone. However, when used ex vivo, the nonspecific toxicity was overwhelming [485]. Meanwhile, when venetoclax was administered ex vivo after bryostatin-1 stimulation, there was insignificant clearance of infected cells, but when venetoclax was combined with HIV-specific CTLs, there was a modest decrease in the amount of HIV-infected cells and when anti-CD3/CD28 was used instead of bryostatin-1, there were significant decreases of the infected cell counts [486]. These data thus support the use of BCL-2 antagonists, but also show that more potent, safe LRAs are needed for this strategy, because anti-CD3/CD28 stimulation cannot be used in vivo for safety reasons [487,488].

### 6.5. Bone Marrow Transplant for HIV Cure

The only two cases of cured PWH have been the “Berlin patient” [343] and “London patient” [8], both of which were treated with allogeneic bone marrow stem-cell transplantation with donors homozygous for the *CCR5* Δ*32* allele. This resulted in reconstructed immune systems that lacked functional CCR5 coreceptors and thereby conferred resistance to HIV. As much as these two cases bring hope to the world of HIV, they are unfortunately alone. The “Boston patients” tried to recapitulate the results without the *CCR5* Δ*32* allele and resulted in viral rebound [344]. Additional follow-up attempts have also not been successful [489], especially with the emergence of non-CCR5 tropic virus negating the use of the *CCR5* Δ*32* [490] and rebound regardless of reservoir quantification [491]. The overall safety of hematopoietic stem cell transplantation is also a heavy burden that restricts this strategy [489].

### 6.6. Broadly Neutralizing Antibodies for the HIV Cure

The use of broadly neutralizing (bn) antibodies (bnAbs) stem from the study of monoclonal antibodies (mAbs), the first of which being b12, which was isolated from a PWH in the 1990s [492]. bnAbs allow for virus elimination through multiple mechanisms: (i) binding blocks the virus from interacting with host cell receptors and can prevent endocytosis, fusion, or penetration [493]; (ii) aggregation of virions due to antibody binding [493]; (iii) recognition by intracellular TRIM21 and targets to proteosome [494]. Antibodies can also target infected cells for antibody-dependent cellular cytotoxicity, although this is considered a function of non-neutralizing antibodies [495]. About a decade ago, the first bnAbs were isolated from a PWH that displayed potent neutralization specific for the CD4 binding site (CD4bs) of gp120. The primary bnAb studied from this individual, VRC01, neutralized 91% of 190 viral strains representing all major circulating HIV-1 clades; the other two bnAbs from the same individual were not as potent. In comparison, b12 was only able to neutralize 41% of isolates [496]. Other bnAbs have since been identified, with other affinities, e.g., the V3 loop and its glycans, the MPER, and the V1-V2 loop of gp120, and are reviewed in [497]. NHP studies demonstrated efficient protection by various bnAbs against SHIV challenges [498,499,500,501,502,503]. Phase I clinical trials have reported the safety of bnAbs in humans [504,505,506] and follow-up trials have now demonstrated that bnAbs are able to suppress viremia. Additionally, administration of bnAbs 3NBC117 and 10-1074 to PWH during ATI resulted in increased Gag-specific CD8^+^ and CD4^+^ T cell responses, as well as lengthened viral suppression [507]. However, there are caveats to the results: (i) suppression is short lived [508,509,510]; (ii) patients developed anti-bnAb responses [508,509], although they were left susceptible to bnAbs targeting different epitopes; (iii) lackluster prevention of virus via cell-to-cell transmission [511,512,513]. Thus, groups are working to develop bnAb cocktails that will cover multiple epitopes for neutralization to potentially enhance efficacy [514,515], including the development of bispecific [516] and trispecific [439] antibodies. bnAbs have been tested in RMs to either cure or induce functional cure. SHIV-infected RMs were treated with bnAbs 3BNC117 and 10-1074 3 days postinfection. In both groups, viremia was controlled while bnAbs were detectable and rebounded after clearance. Postrebound viral control occurred in half of the RMs [517]. However, treatment at 3 dpi lacks clinical relevancy. Thus, repeated experiments were conducted at 14 dpi and ART-treated RMs were included. Postrebound control was partial, and only one animal controlled below the limit of detection. ART did not result in more postrebound controllers [518]. In both studies, CD8^+^ T-cell depletion resulted in abrogated viral control [517,518], supporting the role of CD8^+^ T cells in viral control. BnAbs have also been combined with other cure agents. bnAb PGT121 was combined with TLR7 agonist GS-9620 and administered to SHIV_SF162P3_-infected RMs on ART (initiated 7 dpi). This resulted in delayed viral rebound, with 45% not rebounding and neither adoptive transfer nor CD8^+^ T-cell depletion demonstrated replication competent virus in those RMs [519]. A follow-up study started ART at 14 dpi to increase clinical relevancy [520], and received a different TLR7 agonist, GS-986, with both bnAbs PGT121 and N6-LS. However, treatments did not result in the same viral control, while still being associated with a delay in viral rebound [520]. Another recent study utilized different combinations of IL-15 superagonist, N-803, with bnAbs 10-1074 and 3BNC117. The combination of N-803 and 10-1074 had an efficacy of postrebound control of 60%, while N-803 + 10-1074 and 3BNC117 had a postrebound efficacy of 75% [521].

### 6.7. HIV Vaccines for HIV Prevention or Therapeutics

An HIV vaccine is still not in our grasp. Of the main roadblocks against the development of an effective HIV vaccine one is that no individual has been known to have established an HIV infection and spontaneously completely cleared the infection. A small fraction of PWH spontaneously control the plasma viremia to below the limits of detection and are known as elite controllers, but the mechanisms of their viral control have yet to be elucidated and replicated in viremic individuals [522]. Furthermore, the elite controllers are not eradicating the virus, thus being only useful as models of a functional cure. To date, HIV vaccine clinical trials were not successful: the STEP trial had to be discontinued after the first interim review found non-efficacy and, in fact, increased the risk for HIV acquisition in those that were already Ad5 seropositive [523]. Another vaccine strategy based on the use of the canarypox vector (ALVAC) surpassed the threshold necessary for 50% vaccine efficacy in phase 1–2 trials [524] and continual analysis after a 12-month booster showed increased IgG binding antibody response rates and CD4^+^ T-cell response rates [525]. Unfortunately, after the advancement to a phase 2b/3 trial, HVTN 702, the interim analysis determined that there was no significant difference between infection rates of those vaccinated and thus determined inefficacious [526]. A third vaccine strategy is the use of mosaic vaccines which are focusing on eliciting a CD8^+^ T-cell-mediated control [527,528]. These vaccines use mosaic proteins, polyvalent antigens formed from peptides of natural sequences determined by computer modeling for the optimal coverage of epitopes [529]. A phase I/IIa clinical trial found that the mosaic Ad26 prime with Ad26 + gp140 boost vaccine regimen was highly immunogenic, eliciting strong binding antibody responses, antibody-dependent cellular phagocytosis responses, and T-cell responses. Further, in RMs they found it produced 66% protection from six SHIV-SF162P3 challenges [528]. This vaccine regimen is now being tested in two clinical trials, HVTN 705 (HPX2008; imbokodo) and HVTN 706 (HPX3002; Mosaico). However, the imbokodo study only provided a 25.2% efficacy estimate against HIV infection and was discontinued after phase 2b [530]. Another vaccine using a recombinant modified vaccinia Ankara-based (MVA-B) vaccine with gp120 and a fused Gag-Pol-Nef polyprotein was found to be well tolerated and increased T cell responses against Gag, but did not change the latent reservoir or time to rebound after ATI [531].

Another vaccine strategy that is based on the use of the RhCMV vector was reported to induce a functional cure in half of the vaccinated monkeys when administered prophylactically; cell-associated DNA was detectable without disease progression [34,532,533]. However, given as a therapeutic, the RhCMV/SIV vaccine did not result in control of infection [30]. To try to improve upon their RhCMV vaccine, Hansen et al. [33] deleted the Rh110 gene to suppress lytic capabilities. This ΔRh110 RhCMV/SIV vaccine enabled viral control and progressive clearance in 59% of vaccinated RMs. Further, 75% of the RMs which cleared viremia were able to control a second challenge 3 years later. An adapted human CMV (HCMV) vaccine was tested in uninfected persons to try to mimic the same unconventional MHC II CD8^+^ T cell responses seen with the RhCMV vector but were unsuccessful [534]. Peptides-based strategies are also used for HIV vaccines [535,536]. Early peptide vaccines were moderately immunogenic in humans [537,538] and lacked protection in most recipients [539]. Currently, the VAC-3S vaccine utilizes a gp41 motif (3S) with the CRM197 carrier protein to induce responses against HIV and increased CD4 restoration and reduced PD-1 expression, suggesting an improvement of T-cell exhaustion [540]. The Vacc-4x is another peptide-based vaccine that uses p24^gag^ domains. Vacc-4x did not result in protection, but viral set point was reduced after ATI [541]. Vaccinations several years later, in the same participants, resulted in decreased viral DNA and maintained a reduced viral set point at ATI, but protection remained elusive [542]. However, not all peptides are used for vaccines and are reviewed in [543]. Enfuvirtide is a synthetic peptide that binds gp41 and blocks fusion with the host membrane and is a current HIV therapeutic [544] and Maraviroc blocks CCR5 binding [545]. More recently, a short HIV fusion inhibitory peptide with a longer half-life was developed, IBP-CP24, to overcome the half-life and resistance issues of Enfuvirtide. IBP-CP24 shows promising results with a half-life 14-fold greater than enfuvirtide while reducing plasma viremia in HIV-infected humanized mice [546]. At the extreme end of vaccination are two vaccination strategies: (i) an engineered herpesvirus that expresses all nine SIV gene products [547]; (ii) vaccine regimen with DNA, modified vaccinia Ankara, VSV, Ad5, RM rhadinovirus, and DNA a second time, to achieve vaccination containing the entirety of the SIV Env [548]. From the herpesvirus vaccine, 4/6 animals were protected through six intravenous challenges within four months, supporting future investigation [547]. Unfortunately, the sequential proteome-based vaccine method was unable to elicit protective immunity against SIVmac239, demonstrating the difficulty of SIV/HIV vaccines [548].

### 6.8. Targeting Tregs as a Strategy for Cure Research

**Figure 4:**
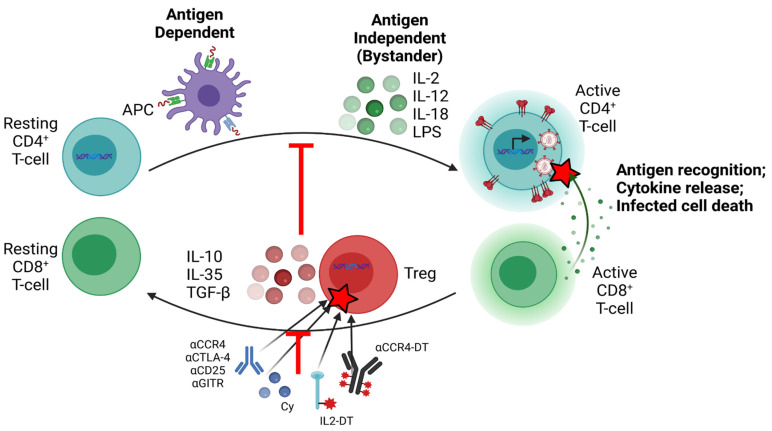
Treg depletion decreases suppressive signaling and allows for T cell activation and latency reactivation. Through the production of various cytokines and immune checkpoints, Tregs suppress other immune cells towards the resting state. By depleting Tregs with various agents, e.g., IL-2-diphtheria toxin or αCCR4, this should allow for activation of T cells to occur and thereby reactivate latent virus as well as bolster reservoir clearance by no longer impeding the cell-mediated immune responses.

The suppressive function of Tregs during HIV infection and the correlations between Treg frequency and HIV/SIV-specific immune responses have led to the strategy of Treg depletion and manipulation. Tregs can be latently infected with HIV and represent a potentially important HIV reservoir: (a) they expand in blood and tissues during chronic HIV and SIV infections [549]; (b) The Treg fraction containing HIV/SIV DNA is higher than in non-Tregs in PWH on ART [550] and RMs [551]; (c) Tregs are less susceptible to cell death than conventional T cells [549]. Meanwhile, during acute infection, Tregs may decisively contribute to the rapid seeding of the HIV reservoir by reversing CD4^+^ T cell immune activation. Finally, during chronic HIV/SIV infection, multiple lines of evidence support a Treg involvement in suppressing protective effector immune responses against HIV: (a) Treg expansion correlates with loss of CTL function [552,553,554]; (b) ex vivo Treg depletion from blood and LNs enhances T-cell responses to HIV/SIV antigens [549]; (c) HIV nonprogressors have a high perforin/FoxP3 ratio; (d) HLAB27^+^ and B57^+^ HIV-specific CD8^+^ T cells from controllers evade Tregs [555,556]. With the suppression of HIV/SIV-specific immune responses by Tregs, and the potential impact it may have on “shock and kill” efficacy, Treg depletion/manipulation may represent a strong cure strategy. Here, through a single intervention, we could directly reduce the reservoir size (via Treg killing), reactivate the virus, and boost cell-mediated immune responses (Figure 4).

However, Treg depletion has its own issues because Tregs are beneficial through the suppression of general immune activation. Additionally, because the best marker for Tregs, FoxP3, is intracellular, targeting in vivo requires the use of less specific markers. Nonetheless, there are several other targets that have been tested for Treg depletion, such as: targeting CD25, CCR4, and GITR, while low dose cyclophosphamide is used through a different mechanism.

#### 6.8.1. Targeting CD25

Denileukin difitox (ONTAK), IL-2-diphtheria toxin conjugate, has been used to deplete Tregs [557,558]. IL-2 binds to its receptor, CD25 which is enriched on Tregs, allowing the conjugated diphtheria toxin to induce cell death after entry [559]. In cancer patients Ontak showed some efficacy [560,561,562,563,564]. In SIVsab-infected RMs, Ontak depleted ~80% of circulating Tregs and resulted in massive increases in T-cell activation, bolstered SIV-specific immune responses, and viral reactivation [441]. A follow-up study in SIVsab-infected RMs with a new anti-human, bivalent IL-2-DT [565,566] demonstrated similar positive results of depletion of peripheral Tregs, including partial depletion in the lymph nodes (>50%) and intestines (25%), immune activation, viral rebound up-to 10^3^ vRNA copies/mL, and bolstered SIV-specific CD8^+^ T cell responses [442]. However, when bivalent IL-2-DT was given to functionally cured RMs on ART, there were severe adverse effects necessitating the suspension of treatment [442]. Further, the specificity of Treg depletion was substantially hindered, with CD4^+^ and CD8^+^ T cells being greatly depleted, and there was no viral reactivation despite the immune activation [442].

#### 6.8.2. Targeting CCR4

Tregs express high levels of CCR4, especially compared to conventional T cells [567,568,569], and can be used as a coreceptor for HIV [570]. Similar to IL-2-DT, an anti-human CCR4-DT conjugate immunotoxin was tested in RMs. Treatment resulted in limited depletion of Tregs relative to IL-2-DT, with greatly limited depletion in the lymph nodes [571]. The anti-CCR4 monoclonal antibody, mogamulizumab, also showed promise against CCR4^+^ malignant cells and CCR4^+^ Tregs [572,573]. Unfortunately, the use of anti-CCR4 immunotoxin in functionally-cured RMs did not result in viral reactivation, regardless of prominent immune activation and proficient Treg depletion [442].

#### 6.8.3. Targeting GITR

Glucocorticoid-induced tumor necrosis factor receptor (GITR) is a member of the tumor necrosis factor receptor family and is expressed on T lymphocytes [574]. The main function of GITR is to protect T lymphocytes from activation-induced cell death [575]. Tregs express more GITR on their surface than non-Tregs and signaling through GITR for Tregs results in suppression of activity [576], different from non-Tregs which results in activation [577,578]. Therefore, anti-GITR antibodies have been explored for the possibility to deplete Tregs and inducing both indirect and direct T-cell activation [579]. In HIV-infected cells, it has been demonstrated that GITR signaling protects infected cells from apoptosis [580]. Thus, although GITR targeting has primarily been established for cancer immunotherapy with some promising results [579,581,582], this can readily be applied to PWH.

#### 6.8.4. Cyclophosphamide (Cy) for Treg Depletion

Cy is a long-standing chemotherapeutic agent, which acts as a nonselective cytoreductive agent in normal chemotherapeutic doses [583,584,585,586]. In low, metronomic dosages, Cy maintains its antitumor properties, but results in decreased adverse effects while improving responses to treatment [587]. Additionally, the low dose treatments result in selective depletion, as well as reduction of suppressive function, of Tregs [588,589,590]. Treg selectivity is attributed to decreased DNA repair and decreased production of glutathione, which eliminates acrolein toxicity [591]. In persons treated with low dose metronomic Cy for four total weeks, Treg depletion occurred and resulted in bolstered cell-mediated cytotoxicity [592]. However, in SIVsab-infected RMs, low dose Cy did not result in acceptable Treg specificity, nor did it greatly induce T-cell activation nor viral reactivation [593]. With additional toxicity occurring when Cy is combined with ART [593], Treg depletion via Cy loses feasibility.

### 6.9. T-Cell Exhaustion and Targeted Therapies

Tackling the immune system dysregulation in PWH is an important goal of any cure approach. During HIV infection, T cells, especially CD8^+^ T cells, gradually lose their effector functions, in a state known as T cell exhaustion, which was first described in chronic lymphocytic choriomeningitis virus-infected mice [594]. In fact, in HIV progressors, the basal phosphorylation levels of proteins downstream from T cell receptor signaling were increased and correlated with impaired signaling [201]. These cells are marked by expression of immune checkpoint molecules, such as cytotoxic T lymphocyte-associated molecule-4 (CTLA-4) [200], programmed cell death-1 (PD-1) [196,197,198,199], T-cell immunoreceptor with immunoglobulin and ITIM domains (TIGIT) [595], and lymphocyte activation gene-3 (LAG-3) [596], as well as glycoprotein T-cell immunoglobulin and mucin domain-containing molecule 3 (Tim-3) [315,597]. PD-1 and LAG-3 expression levels are even correlated with time to virus rebound after ART cessation [598].

#### 6.9.1. CTLA-4 Blockade in HIV Cure

CTLA-4^+^ CD4^+^ T cells are enriched for replication-competent virus [200]. During HIV infection, CTLA-4 prevents proper stimulation of HIV-specific immune cells, thereby protecting infected cells [599,600]. In PWH treated with Ipilimumab (α-CTLA-4 mAb), there was minimal effects on viremia, although single copy assay demonstrated a trend of decreased viral production [443]. In chronically infected animals treated with CTLA-4 blockade, similar decreases in viremia were noted, but there was also increased SIV-specific immune response [444], indicating therapeutic effects of CTLA-4 blockade. CTLA-4 blockade in ART-treated RMs increased T-cell activation and viremia, but did not augment responses to vaccination, nor increase SIV-specific responses [601]. This is contrary to a mouse study which demonstrated increased HIV-specific B-cell and T follicular helper responses with CTLA-4 blockade combined with HIV virus-like particle vaccination [602]. Thus, the evidence points towards a potential usage for CTLA-4 blockade as an immune boosting agent when combined with an LRA, as standalone CTLA-4 blockade does not potently reactivate virus.

#### 6.9.2. PD-1 Blockade in HIV Cure

PD-1 expressing CD4^+^ T cells during HIV infection are also enriched for inducible virus and blockade with nivolumab (anti-PD-1) administered to a patient resulted in increased cell-associated unspliced RNA, yet not plasma viremia, consistent with slight latency reversal [199]. A PWH with advanced nonsmall cell lung cancer was given nivolumab and monitored. The patient had insignificant changes in the plasma viral loads, with an increase in cell-associated DNA which normalized one month later. Overall immune activation markers remained stable, although there were increases in IFN-γ^+^ CD8^+^ cells and cell counts [603]. More promising data emerged from another nivolumab-treated lung cancer PWH. In this individual, plasma viremia demonstrated latency reactivation beginning at D14, while HIV DNA was decreased, and concomitant immune activation increased along with HIV reverse transcriptase and Nef-specific CD8^+^ T cells as well, thus pointing towards a shock and kill mechanism [604]. Unfortunately, the effects of PD-1 blockade are simply inconsistent between patients, as another study demonstrated divergent data from the others, with no consistency between changes in cell associated DNA, RNA, or plasma viremia, as well as the HIV-specific immune responses [605]. However, ex vivo treatment of pembrolizumab (monoclonal anti-PD-1 antibody) with the latency reversing agent bryostatin was able to drastically, and significantly increase the amount of virus induction [606]. The ectonucleotidase CD39, which converts ATP to AMP (subsequently converted to the immunosuppressive adenosine by CD73) [607], is used to identify terminally exhausted CD8^+^ T cells, which are often coexpressing PD-1 [608]. In CD39^+^ CD8^+^ T cells, the adenosine receptor, A2aR is expressed at higher levels in PWH, especially in the treatment naïve. Further, in vitro combination inhibition of PD-1 and A2aR was synergistically more effective than either inhibition standalone in rescuing CD8^+^ T cell function [609]. Thus, CTLA-4 and PD-1 blockades are likely to be most helpful when used in combination with other therapies and/or more potent LRAs.

#### 6.9.3. IL-15 for HIV Cure

IL-15 is associated with the generation and survival of CD8^+^ T cells [610,611], including HIV-specific CD8^+^ T cells and is investigated as an ART alternative or enhancement [612]. IL-15 enhances NK cell activation and function [613]. In RMs, IL-15 induced proliferation of SIV-specific CD8^+^ T cells but did not increase functionality [614,615]. The combination treatment of IL-15 after latency reversal with vorinostat resulted in increased clearance of infected cells [613]. However, free IL-15 administration can be very toxic [616], leading to the development of safer and more effective IL-15 superagonists. The heterodimeric IL-15/IL-15Rα increased CD8^+^ T cells and NK cells activity and decreased viral RNA in the plasma and LN of SHIV-infected RMs [617]. Different IL-15 modifications have been made, but only N-803 [618] (previously ALT-803), has been tested beyond in vitro due to having the greatest efficacy thus far. N-803 has shown reactivation potential in vitro and primed CD4^+^ T cells for recognition by immune effectors [619]. N-803 has shown improved NK cell activation and functionality in vitro and in HIV-infected humanized mice and protected against HIV challenge when given up-to 3 days after challenge [319]. In ART-naive, SIV-infected RMs, N-803 transiently decreased VLs by 1–2 logs [620] while in two studies, latency reversal occurred in ART-treated RMs, but only with CD8^+^ T-cell depletion, which reactivates virus on its own [621,622]. Indeed, in ART-treated SHIV-infected RMs, N-803 did not reactivate latent virus on its own and there was no change in viral DNA, even with immune activation [623]. Thus, N-803 would benefit from combinatorial treatments with a latency reversing agent for reservoir clearance.

#### 6.9.4. IL-21 in HIV Cure

Similar to IL-15, IL-21 expression in SIV/HIV is associated with maintaining the NK, B, and T cell responses [624] and enhances effector functions when given in vitro and ex vivo without large increases in general immune activation [625,626]. Administered to SIV-infected RMs, IL-21 increased CD8^+^ T cell and NK cell activity and increased SIV-specific antibodies in the serum, without inducing CD4^+^ T cell activation nor viral reactivation [627]. In SIV-infected RMs on ART, IL-21 improved intestinal CD4^+^ T cell restoration, with reduced immune activation in both the gut and circulation. After ART cessation, immune activation and plasma VLs remained lower than control animals demonstrating a greatly positive effect of IL-21 treatment [628]. IL-21 in conjunction with IFNα in ART-treated, SIV-infected RMs drastically improved NK cell functionality and Env-specific activity. Further, the treatments resulted in reduced replication competent virus in LNs and increased time to rebound after analytical treatment interruption [629] supporting further investigation of IL-21 treatments.

### 6.10. “Shock and Kill”—Latency Reactivation for HIV Cure

The “shock and kill” approach has become the most widely explored HIV cure strategy with several different classes of agents being tested as potential LRAs: histone deacetylase inhibitors (HDACis) [630,631,632], protein kinase C (PKC) agonists [633,634], ingenol derivatives [635,636,637,638], bromodomain inhibitors [639], second mitochondrial activator of caspases (SMAC) mimetics [640], stimulator of interferon genes (STING) agonists [641], and Toll-like receptor (TLR) agonists [642,643,644,645,646]. This strategy utilizes LRAs to induce viral transcription and replication, which potentiates antigen recognition by immune surveillance and allows immune effectors to eliminate the productively infected cells, theoretically resulting in the reduction of the viral reservoir.

#### 6.10.1. HDAC Inhibitors for “Shock and Kill”

Theoretically, HDACi are strong candidates for latency reversal. Nucleosomes are a basic structural unit of DNA that contain chromosomal DNA wrapped around two of each core histone, H2A, H2B, H3, and H4, forming an octameric core. As the DNA wraps around the cores, it can be modified with acetylation, methylation, and phosphorylation, which changes the binding tightness through charge, thereby affecting the function [647]. In the case of acetylation, histone acetyl transferases (HATS) acetylate the positively charged lysine residues of the histone N termini. This epigenetic change decreases the electrostatic affinity between the histone proteins and DNA and as a result, the DNA becomes more accessible to transcription factors [648,649,650].

**Figure 5:**
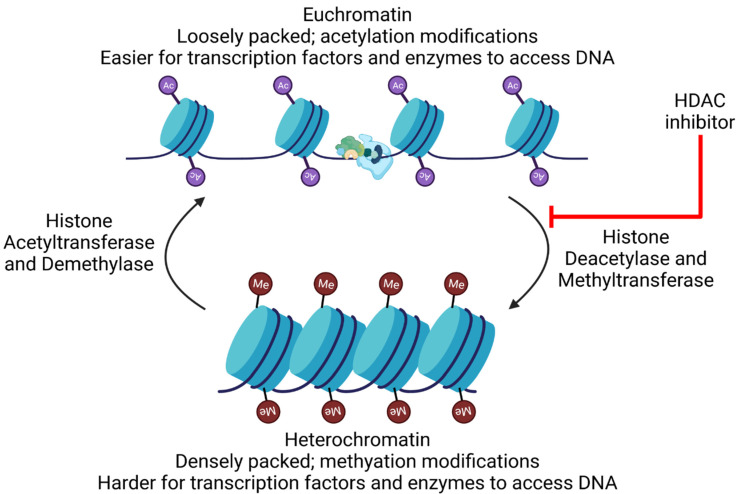
HDAC inhibitors drive chromatin towards a euchromatin state. The HIV LTR is associated with a closed, heterochromatin state suppressing viral transcription by being physically difficult to access. By inhibiting the deacetylation of the histones, this changes the charges associated with the histones and provides a looser conformation such that transcription factors and RNA Polymerase II are better able to access the DNA.

Among genome modifications, deacetylation of the integrated HIV proviral structure around the LTR inhibits transcription of the provirus by tightening the DNA around the histone, thereby preventing proper binding of transcription factors and RNA polymerase II, and therefore preventing viral transcription [361,369,651,652,653] (Figure 5). Disruption of deacetylation has been shown to reactivate latent HIV-1 in vitro [630] and in vivo [632,654,655,656,657,658], however, long lasting changes in the reservoir have yet to be achieved.

Romidepsin (RMD), a bicyclic class I HDACi (targets HDACs 1, 2, 3, and 8) [659,660,661], produces the most potent HIV reactivation ex vivo when compared to other HDACi [306] even at plasma concentrations that are lower than what is used for chemotherapy. These data were reproducible in cells from ART-treated PWH ex vivo [306]. In RMs, in vivo administration of RMD resulted in a massive increases in T-cell activation and viral rebound in post-treatment controllers [453]. RMD administration to PWH and RMs on ART also demonstrated T-cell activation and viral reactivation [658,662]. Yet, neither of these studies were able to demonstrate a statistically significant decrease in the SIV/HIV reservoir. Ex vivo, RMD (as well as Panobinostat and vorinostat [SAHA]) was shown to have a negative effect on the HIV-specific immune response, i.e., suppression of cytokine production and decreased cellular viability [451], as well as reduced proliferation and viability and also restriction of de novo infections after stimulation with IL-2 and PHA [663]. Unlike Jones et al. [451], Jønsson et al. showed that RMD treatment differentially changed expression patterns of interferon-stimulated genes, such as increases in IFIT1, ISG15, and STAT1, but decreases in APOBEC3G, MX2, and TRIM22 [663]. In RMs, the SIV-specific immune response was not significantly altered [453], nor were the HIV-specific immune responses altered in PWH in vivo [658]. In the BCN02 clinical trial (NCT02616874), PWH received MVA. HIVconsv vaccination and weekly infusions of RMD, and, while RMD administration reduced the total number of vaccine-elicited T cells secreting multiple cytokines, the CD8^+^ T cells retained their HIV suppressive functionality [664].

#### 6.10.2. Protein Kinase C (PKC) Agonists for “Shock and Kill”

PKC agonists work through the canonical NF-κB pathway to enable HIV reactivation [665,666]. Many of the agents: phorbol esters (prostratin) [633]; bryostatin-1 [667] and its analogs [668,669]; have demonstrated HIV reactivation potential in vitro and ex vivo [670], but the common problem when moving to in vivo models was generalized immune activation and its resulting toxicity/tolerability due to higher doses required for in vivo reactivation [449,671]. To counter this issue, combinatorial LRA treatments, such as bryostatin-1 or prostratin with an HDAC inhibitor (e.g., Romidepsin, SAHA, and largazole) or bromodomain inhibitor JQ1, allow for lower LRA doses with similar or increased potency and decreased toxicity ex vivo [672,673]. Similarly, prodrugs for prostratin, ingenol, and bryostatin-1 were developed to improve tolerability and reduce bolus toxicity, and maintained immune activation and HIV reactivation in vitro and ex vivo [674]. However, these combinations and prodrugs have yet to be tested in vivo and further testing is warranted.

#### 6.10.3. Ingenol Derivatives for “Shock and Kill”

Ingenol 3-angelate is an inflammatory substance extracted from the sap of the *Euphorbia peplus* plant [675]. Ingenol 3-angelate and its derivatives are structurally analogous to phorbol esters and mechanistically act through the PKC and NF-κB pathway [676,677]. By activating the NF-κB pathway, ingenol derivatives reactivate latent HIV in vitro and ex vivo [636,637,677,678]. Initially, ingenol derivatives were investigated for HIV inhibition and CD4 downregulation through PKC activation and data from the same study showed some derivatives reactivated HIV [679]. With data demonstrating that PKC agonists have anti-latency properties, renewed focus was placed upon new ingenol derivatives that can promote HIV reactivation, while reducing the toxicity associated with early ingenol derivatives, PMA, and prostratin [678,680]. The derivative ingenol-B reactivated HIV in vitro [681], but in ART-treated, SIV-infected RMs, ingenol-B did not reactivate virus in the circulation, but did have a viral blip in the cerebrospinal fluid (CSF). In combination with the HDACi vorinostat, reactivation was achieved in circulation and CSF [173]. In PWH treated with ingenol mebutate gel on the skin, HIV was reactivated locally in skin biopsies without plasma viremia, nor systemic immune activation [682]. The intravenous version of ingenol mebutate, PEP005, was found to have a synergistic reactivation when combined with JQ1, while also downregulating surface receptors CD4, CCR5, and CXCR4 ex vivo [636]. Thus, further research is warranted.

#### 6.10.4. Bromodomain Inhibitors for “Shock and Kill”

JQ1, a small molecule bromodomain inhibitor developed in 2010, was shown to bind to BRD4 [683]. BRD4 competes with Tat for P-TEFb binding, restricting HIV replication [684]. JQ1 treatment of HIV-infected CD4^+^ T cells modestly reactivated virus, while suppressing T cell proliferation and downregulating CD3, CD28, and CXCR4 in vitro and ex vivo with minimal toxicity [639]. The combination of JQ1 with prostratin produced synergistic increases in reactivation in vitro [388]. Due to the modest reactivation potential of JQ1, new bromodomain inhibitors have been tested, with OTX015 [685], UMB-136 [686], apabetalone [687], and CPI-203 [688] showing greater reactivation potential, while also maintaining minimal toxicity and the synergistic activity with PKC agonists (e.g., prostratin and bryostatin-1) in vitro. Additionally, 8-methoxy-6-methylquinolin-4-ol (MMQO) [689], a quinolone based bromodomain inhibitor, reactivates HIV ex vivo and maintains immunosuppression similar to JQ1, without acting through the Tat transactivator [690]. Thus, these different bromodomain inhibitors are promising LRAs, but require in vivo studies to further elucidate their potential.

#### 6.10.5. Second Mitochondrial Activator of Caspases (SMAC) Mimetics for “Shock and Kill”

SMAC mimetics are of high interest as LRAs because: (i) BIRC2 acts as a repressor of the NF-κB pathway and is antagonized by SMAC mimetics [691,692]; (ii) less generalized immune activation through the noncanonical NF-κB pathway versus canonical pathway [693]; (iii) reactivates HIV in vitro and synergizes with HDACis [694]. The SMAC mimetics AZD5582 [640] and birinapant [695] have both shown reactivation potential in vitro and ex vivo. AZD5582 was also shown to induce viral reactivation in BLT humanized mice and RMs with minimal systemic immune activation, no reduction in CD8^+^ T-cell immune responses, and low toxicity [696]. AZD5582 also has greater reactivation potency when combined with CD8^+^ cell depleting antibody M-T807R1 [697]. The SMAC mimetic Ciapavir demonstrated similar functionality in humanized mice and synergized with bromodomain inhibitors JQ1 and I-BET151, but when used with bryostatin-1 or ingenol-3-angelate induced greater toxicity [698]. SMAC mimetics are also showing efficacy against infected macrophages, but this is discussed later. Overall, SMAC mimetics warrant additional exploration in vivo.

#### 6.10.6. Stimulator of Interferon Genes (STING) Agonists for “Shock and Kill”

STING agonists were of initial interest as boosters for the innate immune response and antigen-specific immune responses [699]. Indeed, one STING agonist, 3′3′-cGAMP primed HIV-1-specific CD8^+^ T cells [700] and cGAMP delivered with nanoparticle PC7A induced protection against HIV through type I IFN and inhibited HIV-1 replication [701]. However, cyclic GMP-AMP (cGAMP) and c-di-AMP are also capable of latency reactivation ex vivo while also increasing the frequency of SIV-specific CD8^+^ T cells [641]. In an ex vivo combinatorial study, the STING agonist cGAMP and HDACi, resminostat, had additive, but not synergistic reactivation potential [702]. In a pilot study of ART-treated, infected RMs, the STING agonist reactivated SIV in a third of RMs [703]. Thus, STING agonists are promising LRAs, but will be much more effective in combinatorial regimens.

#### 6.10.7. Toll-like Receptor (TLR) Agonists for “Shock and Kill”

TLR agonists are similar to STING agonists in that they were initially looked at for HIV inhibition [704], yet are now investigated as both LRAs and immunomodulatory agents. Because TLRs are pattern recognition receptors they react to signals indicating the necessity for an immune response [705]. TLR2 and TLR9 agonists reactivated HIV from transgenic mouse spleen cells ex vivo [706]. Although that was the first major study to demonstrate and explain the reactivation, a previous clinical trial for the antisense oligodeoxynucleotide phosphorothioate GEM91 [707] had noted increased HIV-1 [708], contradictory to the ex vivo data with GEM91 [704], and it is hypothesized that this was through TLR9 activation [708]. Further investigation into a TLR9 agonist, CPG7909, as a pneumococcal vaccine adjuvant in PWH showed increased immunogenicity, a boost in HIV-specific CD8^+^ T cells and a reduction in caDNA, but also increased adverse effects [709]. MGN1703 was thus developed to decrease the toxicity of existing TLR9 agonists [710] and was tested ex vivo [711] and then in PWH with 4 weeks [712], followed up with a second study of 24 weeks of treatment [713]. Although MG1703 resulted in increased innate immune responses and HIV reactivation, overall viral burden was not significantly reduced, nor was a there difference in time to rebound [713]. TLR7 agonist GS-9620 reactivated HIV ex vivo and enhanced HIV-specific CD8^+^ T cells [310]. These results were seconded in SIV-infected RMs, with reduced caDNA and inducible virus after TLR7 agonist GS-986 treatment [35]. However, GS-9620 was unable to reproduce any of the virological effects of GS-986 in RMs [714] nor PWH [715]. Combinatorial TLR2 (Pam2CSK4) and TLR7 (GS-9620) agonists were tested ex vivo and enhanced reactivation potency by acting through separate mechanisms, thus suggesting this strategy should be tested further in vivo to improve outcomes versus single TLR agonist administration [646].

### 6.11. When Calories Get Serious—Role of Immunometabolism in HIV Pathogenesis and Cure

#### 6.11.1. Immunometabolism and HIV Pathogenesis

Immunometabolism refers to the interface between the previously distinct fields of metabolism and immunology. With time, researchers have clearly shown that these are in fact linked, with specific metabolites being required for a proper function of macrophages, neutrophils and T cells, such as glucose, glutamine, fatty acids, and amino acids [716]. As for the pathways involved, there are six utilized in immune cells: glycolysis (the main metabolic pathway for T cell effector functions [717]), tricarboxylic acid (TCA) cycle, pentose phosphate pathway (PPP), fatty acid oxidation (FAO), fatty acid synthesis (FAS), and amino acid metabolism. Additionally, mTOR is an important regulator in the adaptive immunity, especially for the CD8^+^ T cell response [718]. However, immunometabolism differs between acute, chronic, and latent viral infections. During acute infection, when the CD8^+^ T cells are developing they upregulate mTORC1 and aerobic glycolysis to support their energy demands. However, during this time, HIV-specific T cells are beginning their metabolic dysregulation with extensive proliferation and activation, promoting an altered mitochondria that is burnt out to sustain the hyperproliferative state [719]. With chronic infection, T cell exhaustion and T cell metabolism become highly correlated, such as PD-1 ligation which results in diminished glucose and amino acid metabolism and mTOR activity [720,721,722]. Differently from PD-1, CTLA-4 modulates glycolysis and amino acid metabolism, but does not enhance FAO as seen with PD-1 ligation [720], a mechanism which is thought to promote survival during PD-1 ligation when the other metabolites are not being utilized. During chronic viral infections CD8^+^ T cells were profiled with glycolysis dependency, dysfunctional mitochondria, and abrogated oxidative phosphorylation (OXPHOS), which is involved in the TCA cycle [723]. Interestingly, the same study demonstrated that CMV-specific T cells were more functional and able to utilize OXPHOS, but the glycolysis pathway was inhibited. This points towards differences in the metabolism of not only chronic versus latent infections, but also of functional CD8^+^ T-cell responses [723]. In HIV controllers, CD8^+^ T cells were found to be metabolically separate from progressor CD8^+^ T cells. HIV controller CD8^+^ T cells were characterized by the upregulation of survival genes pathways and metabolic plasticity, (with functional mitochondria and OXPHOS) and is supported by the mTORC2 pathway [724].

#### 6.11.2. Modulation of Immunometabolic Programming for HIV Cure/Therapeutics

With the complex relationship between HIV/SIV pathogenesis, immunometabolism, and T-cell exhaustion, there is growing interest in targeted metabolic therapies. Another effect of metabolism is the susceptibility to HIV. The accepted paradigm is that CD4^+^ T-cell susceptibility to HIV increases with differentiation, but what was recently described is the propensity for HIV to selectively infect CD4^+^ T cells that are utilizing high levels of OXPHOS and glycolysis. Partial in vitro *i*nhibition of glycolysis with 2-deoxy glucose (2-DG) demonstrated that the glycolytic environment is required to complete reverse transcription and had a greater effect in further differentiated cells. Further, limiting glycolysis with 2-DG also showed a selective toxicity towards infected cells and 2-DG was also able to greatly reduce HIV replication after phytohemagglutinin (PHA) stimulation [725], thereby showcasing an additional metabolic regulation of HIV that can potentially be exploited. Regarding dysfunctional mitochondria, IL-12 administration was able to reverse the dependence on glycolysis and restore mitochondrial changes and metabolic pathways [723]. IL-15 is also known to promote FAO and mitochondrial biogenesis [726]. Ex vivo, CD8^+^ T cells from noncontrollers were shown to be enhanced after IL-15 treatment, with increased fatty acid uptake and enhanced mitochondrial respiratory capacity. Further, IL-15 pretreatment enhanced the SIV-specific CD8^+^ T-cell response of SIV-infected macaques and restored metabolic plasticity [724]. These two studies thus point towards potential therapeutics for HIV via metabolic restoration.

### 6.12. True or False—Macrophages and the HIV/SIV Reservoir

#### 6.12.1. Macrophages Contribution to the HIV/SIV reservoir

In addition to the CD4^+^ T cells, macrophages also harbor provirus and are capable of producing replication competent virus. In macaques infected with SHIV_DH12R_ (highly pathogenic SHIV containing envelope glycoproteins from HIV-1 strain DH12 [727]), following the characteristic extensive depletion of the CD4^+^ T cells, the remaining virus-producing cells were 95% macrophages with less than 2% expressing the CD4 receptor [728]. Although studies have not been able to concretely agree on the presence of replication competent virus in peripheral blood monocytes, the presence of HIV in tissue macrophages is undeniable [312,729,730,731,732]. HIV studies utilizing humanized myeloid-only-mice (MoM) demonstrated the ability of macrophages to maintain infection without CD4 cells [418]. Further, ART administration to infected MoM after infection resulted in two-thirds of the MoM from developing persistent infection with one third of the treated mice developing persistent infection that allowed for viral rebound after ART cessation, thus demonstrating the ability of HIV to persist in macrophages and reconstitute infection after ART [733]. Unfortunately, HIV infection of macrophages does not lead to viral cytolysis or apoptosis, with macrophages resistant to Vpr-mediated apoptosis [734].

HIV-infected macrophages are particularly present in the brain and central nervous system (CNS) [730]. During acute infection, infiltration of CD4 cells and monocytes from the blood to the brain allows for infection of the microglia and perivascular macrophages, causing neurological disorders, such as asymptomatic neurocognitive impairment, mild neurocognitive disorder, and HIV-associated dementia [735]. Fortunately, ART reduces neurological disorders. In brains of ART-treated and untreated PWH with HIV-associated neurocognitive disorders, genome-wide microarray analysis found that ART reduced the dysregulation of the brain transcriptome relative to untreated individuals, but regardless of ART, there was still a portion of adaptive and innate immune response genes that were upregulated in both treated and untreated.

#### 6.12.2. Crosstalk between Macrophages and Exhausted T Cells

The exhaustion of T lymphocytes during HIV infection may provide further detriment to the host than lack of reservoir clearance. During infection, killing of HIV-infected CD4^+^ T lymphocytes by CTLs is a major mechanism of viral suppression. However, the elimination of macrophages is a harder task to accomplish. In SIV-infected macaques, CD8^+^ T cells ex vivo were unable to eliminate infected macrophages to the same extent as they could CD4^+^ T cells [736]. Although they are not the primary reservoir for HIV/SIV, the interaction between CTLs and infected macrophages yields a new dilemma: the extended formation of the synapse induces further secretion of IFN-γ and other pro-inflammatory cytokines [737], thereby potentially increasing the chronic inflammation during HIV infection. Similarities to this are seen in dendritic cell: T cell interactions [738], and the priming of naïve CD8^+^ T cells is demonstrated to alleviate the lack of killing [739,740].

#### 6.12.3. Targeting Macrophages with “Shock and Kill”

Although most of the “shock and kill” therapeutics are aimed at CD4^+^ T cells, recent research shows that SMAC mimetics LCL-161, AT-406 (also known as Debio-1143), and birinapant, can also play a role in the direct elimination of HIV-infected macrophages. With an upregulation of BIRC2 and XIAP in HIV_BA-L_-infected macrophages, similar to HIV-infected CD4^+^ T cells, the infected macrophages are 10-100x more susceptible to cell death via SMAC mimetic than uninfected macrophages [741]. Of the three mimetics tested, only AT-406 resulted in viral reactivation in the macrophages [741], supporting previous findings that AT-406 induces viral reactivation in resting CD4^+^ T cells from PWH and humanized mouse models with ART [742]. Thus, SMAC mimetics are looking to be promising new LRAs.

## 7. Further to Fly: Future Perspectives for HIV Cure and Avenues to Explore

During progressive HIV infection, a healthy immune response is not present, and agents aimed at improving the immune system of PWH have not recapitulated the viral control observed in our model of RM functional cure [71,72] when used alone. Additionally, LRAs were shown to only reactivate a small portion of reservoir [400,448,743], regardless of cellular activation status [744] and have yet to demonstrate substantial reductions in the latent reservoir. As such, these results further diminish the usefulness of single therapy regimens, as the vast majority of the reservoir will remain untouched because the lack of viral production will prevent recognition by the immune system, let alone the lack of clearance by viral cytopathic effects. Nonetheless, the silver lining in current HIV cure research is that recent combinatorial studies have resulted in much better efficacy than the single treatment studies [521,613,636,702]. However, these studies have been completed either during acute infection or with ART initiation during acute infection, and are thus not as applicable to the majority of PWH. Regardless, those studies point towards the eventual development of a functional cure being possible through bolstering the immune response while limiting extensive viremia. Moving forward, the field will need to continue to rely heavily upon NHP models for HIV cure due to the intricacy of combinatorial studies and, more importantly, the potential for unexpected adverse effects. For example, the use of IL2-DT for Treg depletion had a positive impact on the spontaneously functionally cured SIV infection in RMs [441], but when combined with ART, resulted in unacceptable toxicity [442]. Similarly, Cy used as a cytoreductive agent close to chemotherapeutic dosage with ART resulted in unacceptable toxicity and morbidity [593]. Further, the venetoclax + ixazomib combination had synergistic in vitro efficacy, but did not make it to in vivo testing because ex vivo toxicity with PBMCs was too great [485]. Nevertheless, new combinations must still be tested and given the parallels between cancer and HIV, particularly in relation to the role of immune dysfunction in both disease paradigms, it is imperative that we work closely together to achieve greater results [745]. In addition, the field should take note from its own research on ART and try to find combinations that target different mechanisms of HIV latency persistence or immune dysfunction, similar to how there is an antiretroviral for each step of the HIV life cycle. This is because, as demonstrated in every study, what works for one animal or patient may not work for another and this needs to be taken into consideration. By trying different combinations, we may end up with separate treatments for separate phenotypes or stages of infection, e.g., PWH that initiated ART late during chronic infection may respond better with the inclusion of a PD-1/PD-L1 or CTLA-4 blockade due to the further extent of T-cell exhaustion. Meanwhile, during acute infection, a combination of IL-15/21 or TLR agonist with bnAbs may be more efficient in boosting the innate and adaptive immune responses while decreasing excessive viremia and immune depletion, allowing for the cell-mediated immune response to have a better response to virus. Because researchers control when the animals are infected and begin antiretroviral therapy, the NHP models are a wonderful resource for testing these hypotheses.

One of several strategies worth investigating is combining TLR2 and TLR7 agonists. Dual TLR2/TLR7 agonists resulted in increased efficacy through separate immune activation mechanisms [646], and may work better than single TLR7 agonists for viral reactivation and immune stimulation. Further, combination of TLR2/7 agonists with bnAbs [519,520] may be a valid strategy for clearance after reactivation and maintaining immune control, similar to the GS-9620 + bnAb PGT121 [519] or IL-15 superagonist (N-803) + bnAbs 10-1074 and 3BNC117 [521] studies. Interestingly, a cancer study showed that HDACis increase production of human endogenous retroviral elements in cancer cells and that concomitant treatment of TLR7/8 agonists allows for the cells to induce intrinsic apoptosis when they otherwise would not have enough stimuli to do so, while also at doses that are individually subcytotoxic [746]. This therapy can readily be applied to HIV research, but will first require ex vivo testing to ensure cell viability of uninfected cells is maintained and bystander death is not prolific. Should toxicity prove acceptable, additional inclusion of bnAbs could assist by providing another mechanism of reservoir clearance and would not modulate immune activation, thereby avoiding an increase in the chance of cytokine storm. However, as HDACi do not reactivate a substantial portion of the reservoir [400,448,743], repeated treatments would likely be necessary with advancement to in vivo testing.

Other combinations for improving the immune function should also be investigated. IL-15 and IL-21 have both shown efficacy for improving viral control [319,627,628] but have yet to be tested in combination for HIV cure. In mice, IL-15 and IL-21 both act on B, T, and NK cells, but IL-15 is essential for T cells, whereas IL-21 is more important for NK cells. Indeed, there is still overlap between the two and they act synergistically to boost CD8^+^ T cells and function [747,748] and, importantly, improve antigen-specific T-cell responses [749]. Thus, a study of the combination of IL-15/21 as an HIV therapeutic is warranted ex vivo and in vivo, although this combination would likely require substantially decreased doses relative to single treatments to prevent cytokine storms. IL-15/21 would likely also not be feasible as a combination with a LRA, due to too much immune stimulation, but could work very well with bnAbs during acute or early chronic infection. One downside to IL-15 therapy is the induction of PD-1 and PD-L1 expression [750] and unsurprisingly, IL-15 superagonist N-803 is currently being tested with PD-1 monoclonal antibodies to reduce the increased PD-1/PD-L1 expression while also inherently decreasing T-cell exhaustion for cancer treatments [751,752,753]. Another method of combination IL-15 or IL-21 and αPD-1 is the use of fusion proteins. By placing IL-15 [754] or IL-21 [755] on a PD-L1 or PD-1 antibody, IL-15/21 are targeted to PD-1 expressing cells, which will be enriched for tumor reactive CD8^+^ T cells. This can also apply to HIV-specific CD8^+^ T cells which are likewise enriched for PD-1/PD-L1 [196,197,198,199] and may reduce the risk of inducing systemic immune activation with combination therapy. Both of these concepts deserve further investigation. Further, as neither IL-15, IL-21, nor PD-1 therapies have strong latency reversal potency, in vitro experiments should be carried out with the addition of a stronger LRA. In fact, the combination of a SMAC mimetic (i.e., ciapavir) with a bromodomain inhibitor (i.e., JQ1 or a newer molecule) could be a strong contender, as these two drugs have been shown to be synergistic for latency reversal [698]. This particular combination is preferable for a combination with IL-15 or 21 because both ciapavir and JQ1 induce viral reactivation with minimal immune activation, and thus this should limit the potential adverse effects from systemic immune activation. Nonetheless, NHP modeling should be utilized prior to administration to human study participants to elucidate the extent of adverse effects.

Overall, there are many developing combinatorial strategies that are increasing our hope for an HIV cure or remission. In the meantime, we will continue to rely on ART, one of the most successful therapeutic approach of the 20th century. Similar to Paul and Art, HIV and ART are an exquisite combination and had a tremendous and sustained success. In time, cure research will do exactly what Paul Simon succeeded to do so well—live and succeed without ART.
